# Photosynthesis: Genetic Strategies Adopted to Gain Higher Efficiency

**DOI:** 10.3390/ijms25168933

**Published:** 2024-08-16

**Authors:** Naveed Khan, Seok-Hyun Choi, Choon-Hwan Lee, Mingnan Qu, Jong-Seong Jeon

**Affiliations:** 1 Graduate School of Green-Bio Science, Kyung Hee University, Yongin 17104, Republic of Korea; naveedkhanchemist@gmail.com (N.K.); shbam@khu.ac.kr (S.-H.C.); 2 Life and Industry Convergence Research Institute, Pusan National University, Miryang 50463, Republic of Korea; chlee@pusan.ac.kr; 3 Department of Molecular Biology, Pusan National University, Busan 46241, Republic of Korea; 4 Jiangsu Key Laboratory of Crop Genomics and Molecular Breeding, College of Agriculture, Yangzhou University, Yangzhou 225009, China

**Keywords:** photosynthesis, genetic engineering, Calvin-Benson cycle, electron transport chain, photorespiration, abiotic stress, non-photochemical quenching, photosystem

## Abstract

The global challenge of feeding an ever-increasing population to maintain food security requires novel approaches to increase crop yields. Photosynthesis, the fundamental energy and material basis for plant life on Earth, is highly responsive to environmental conditions. Evaluating the operational status of the photosynthetic mechanism provides insights into plants’ capacity to adapt to their surroundings. Despite immense effort, photosynthesis still falls short of its theoretical maximum efficiency, indicating significant potential for improvement. In this review, we provide background information on the various genetic aspects of photosynthesis, explain its complexity, and survey relevant genetic engineering approaches employed to improve the efficiency of photosynthesis. We discuss the latest success stories of gene-editing tools like CRISPR-Cas9 and synthetic biology in achieving precise refinements in targeted photosynthesis pathways, such as the Calvin-Benson cycle, electron transport chain, and photorespiration. We also discuss the genetic markers crucial for mitigating the impact of rapidly changing environmental conditions, such as extreme temperatures or drought, on photosynthesis and growth. This review aims to pinpoint optimization opportunities for photosynthesis, discuss recent advancements, and address the challenges in improving this critical process, fostering a globally food-secure future through sustainable food crop production.

## 1. Introduction

The fast-growing global population and shrinking arable land have exacerbated the severity of the food crisis. With the global population projected to reach 9.3 billion by 2050, it is imperative to maximize world food production using the available land resources [1]. This challenge is compounded by climate change effects such as soil degradation, desertification, and salinization. Concurrently, the rise in atmospheric CO_2_ levels intensifies the greenhouse effect, resulting in increased high-temperature stress on agriculture and forestry, underscoring the urgent need for innovative strategies to secure food supplies sustainably [2,3,4].

Increases in crop yield potential depend on increased total biomass. Modern crops are highly efficient at rapidly spreading their leaf canopies to maximize light interception and at partitioning carbon and nutrients into seeds. However, crops are not as efficient at converting absorbed light energy into sugars through photosynthesis. Analysis has shown that the efficiency of current crop cultivars in converting CO_2_ into sugar molecules falls far short of the theoretical maximum, likely due to evolutionary constraints. Natural evolution prioritizes survival over improving efficiency [5,6]. Photosynthesis is one of the most studied plant processes, and studies have yielded key insights into how its efficiency could be increased. This has culminated in substantial increases in photosynthetic efficiency, crop productivity, and sustainability in replicated field trials. These advances are now being applied to key food crops [7]. One such innovation arose from a theoretical model, which described that during transitions between sun and shade, the recovery of photosynthesis efficiency is very slow; tuning this phenomenon might improve biomass by up to 30% [8]. This idea was later experimentally proved by Kromdijk and his colleagues, who tuned the expression of key enzymes involved in qE-dependent non-photochemical quenching [9]. Similarly, another innovation aimed at future-proofing soybeans against rising CO_2_ concentration and temperature has already been demonstrated under field conditions [10]. These studies represent fundamental breakthroughs in understanding and engineering photosynthesis, transitioning from proof of concept to expanded field testing.

However, the implementation of genetic engineering approaches to enhance photosynthesis faces several limitations and challenges, as reviewed recently by Batista-Silvia et al. [11]. The sensitivity of photosynthesis to environmental changes significantly impacts its measurement, as photosynthetic rates can vary drastically even within different periods of the day. This variability is influenced by fluctuating factors such as light intensity, temperature, and humidity, complicating the acquisition of consistent and reliable data. The heterogeneity of environmental parameters and the logistical challenges of transporting photosynthetic equipment in paddy fields further exacerbate these difficulties. Consequently, the effective measurement of photosynthesis in paddy fields is hindered, complicating the exploration of key genetic markers involved in natural variation [12]. Another major challenge is the complexity of the photosynthetic process itself [13,14], which involves numerous genes and regulatory networks [15]. Modifying one component often requires compensatory changes in others, making the engineering process intricate and susceptible to unintended consequences [13]. Furthermore, the off-target effects of gene-editing tools such as CRISPR-Cas9 have not been explored well, which may lead to cleavages at unintended sites and produce adverse side effects [16,17], particularly in plants where the genome is not well characterized [18]. Additionally, there is the issue of gene expression stability across different environmental conditions [19,20] and developmental stages [21], which can affect the consistency of the desired traits. Furthermore, regulatory and public acceptance hurdles pose significant obstacles, as genetically modified organisms (GMOs) are subject to rigorous approval processes and face varying acceptance levels worldwide [22,23]. These challenges necessitate a multidisciplinary approach, integrating advanced genetic techniques with comprehensive field testing and robust regulatory frameworks to ensure the safety and effectiveness of engineered crops.

This review provides the genetic underpinnings of photosynthesis in crops, exploring avenues for boosting photosynthesis efficiency and adaptability under different environmental conditions. Through a comprehensive analysis of current research and technological breakthroughs, this review aims to illuminate the path forward in optimizing photosynthesis, thereby contributing to global food security in the face of escalating environmental challenges. 

## 2. Advances in Genetic Engineering to Optimize Photosynthesis

For decades, plant genetic engineering has been a crucial tool used in plant science to improve yields and resilience. The first genetically engineered plant, a tobacco variant resistant to herbicides, was developed in 1983 [24]. This breakthrough paved the way for further innovations. Since then, innovations in genetic engineering have equipped scientists with tools to tackle previously unexplored problems. These innovations include various gene-editing technologies such as Zinc Finger Nucleases (ZFN), Transcription Activator-Like Effector Nucleases (TALENs), and CRISPR-Cas9. In parallel, synthetic biology approaches, such as virus-induced gene silencing (VIGS), artificial mini-chromosomes (AMChr), and nanoparticle technology, have revolutionized our approach to solving complex agricultural problems, enhancing both crop yields and resilience [25,26,27,28]. Recent research has offered compelling evidence that enhancing photosynthetic systems through genetic engineering might offer a way to increase yield potential. The improvement in photosynthesis has received significant attention as a crop productivity optimization approach [11]. Since most plant species share the same fundamental biochemical processes for photosynthesis, various genetic-modification techniques have been the primary means of enhancing crop photosynthetic efficiency. Moreover, phenotypic diversity seen in wild species and in crop species suggests that genetic variation also exists in photosynthesis, providing a largely unexplored resource for the breeding of crops with enhanced photosynthesis and increased yields. This has not been thoroughly investigated since the variation most likely involves hundreds of genes, each contributing very little to photosynthesis, making them difficult to identify without appropriate phenotyping and genetic techniques [29]. Genetic engineering, in combination with systems/synthetic biology and computational modeling techniques, may be able to address these issues [30,31]. Synthetic and many genetic technologies may readily overcome problems by designing and rebuilding entire processes rather than merely swapping out individual genetic components [32]. Here, we highlighted studies employing molecular genetics to modify various pathways, readily contributing to enhancing photosynthesis. These modifications, achieved through precise genetic interventions, have shown promising results in boosting photosynthetic efficiency.

### 2.1. Modification of Electron Transport Chain

Enhanced photosynthetic carbon fixation and yield can be achieved by manipulating the photosynthetic light reaction [33]. The light reaction begins with capturing photons using light-harvesting antenna complexes, which transfer energy to excite electrons in the chlorophyll-a molecules of the photosystem reaction centers. These excited electrons travel through membrane-embedded redox carriers, starting from the oxygen-evolving complex (OEC) of photosystem II (PSII) and traveling to the cytochrome b6f (Cyt b6f) complex via plastoquinone (PQ). The electrons then move from the Cyt b6f complex to photosystem I (PSI) via plastocyanin (PC). This electron movement facilitates the transfer of protons from the stroma to the lumen, creating a proton motive force across the thylakoid membrane. This force drives the synthesis of adenosine triphosphate (ATP) from adenosine diphosphate (ADP) through chemiosmotic coupling via ATP synthase [34]. The excited electron from PSI is transferred to ferredoxin, where it reduces nicotinamide adenine dinucleotide phosphate (NADP+) to NADPH through the action of ferredoxin-NADP+ reductase (FNR) [34,35] (Supplementary Figure S1).

The first bottleneck of the photosynthesis light reaction is the light capture inefficiency. Notably, over 50% of energy losses in photosynthesis are attributed to the kinetic mismatch between rapid photon capture and slower downstream electron-transfer processes. This inefficiency primarily arises due to the large optical cross-section of light-harvesting antenna complexes. Adjusting the size of these antenna complexes has shown significant potential in enhancing photosynthesis and biomass production. Perrine et al. (2012) demonstrated that reducing chlorophyll b levels in *Chlamydomonas reinhardtii* optimized light capture, subsequently improving photosynthetic efficiency and growth [36]. This strategy was then implemented to higher plants; Friedland et al. (2019) generated several *Camelina sativa* transgenic RNAi plants with varying levels of chlorophyll b reduction by targeting the chlorophyllide a oxygenase (CAO) gene. These transgenic plants exhibited a diverse array of phenotypes, demonstrating the impact of modulating chlorophyll b levels on photosynthetic efficiency and plant growth. Their findings revealed a critical threshold or optimal antenna size with a chlorophyll a/b ratio of about 5, where a further reduction in antenna size adversely affected photosynthesis efficiency, light stress tolerance, and thylakoid membrane architecture. However, plants with optimized antenna sizes performed well in both controlled greenhouse and field conditions, achieving a 40% increase in biomass yield [37]. Moderate reductions in antenna size led to increased photoprotection and efficiency, while excessive reductions impaired photoprotection and photosynthetic performance. Moreover, Wu et al. (2020) identified that, in *Camelina*, a chlorophyll a/b ratio of 5 represents the optimal antenna size, balancing light capture and energy conversion, enhancing photosynthetic efficiency and high-light stress tolerance. They also demonstrated that, for crop plants growing in different environmental conditions, the optimal antenna size will vary depending on the relative level of photo-oxidative stress [38]. 

During the evolutionary transition from cyanobacterial endosymbionts to eukaryotes, there was a significant loss of genetic components accompanied by the acquisition of several novel proteins [39,40]. Major transformations occurred during the shift from cyanobacterial ancestors to chloroplasts in unicellular algae, whereas the subsequent evolution from algae to terrestrial plants involved comparatively fewer modifications. For example, the cytochrome (Cyt) c6 transporter was lost, and plastocyanin was introduced in flowering plants. Additionally, NADH dehydrogenase (NDH), lost in algae, was reacquired in flowering plants. The detailed evolutionary dynamics of the gain and loss of electron transport chain (ETC) components have been comprehensively reviewed [33,39,40].

**Figure 1:**
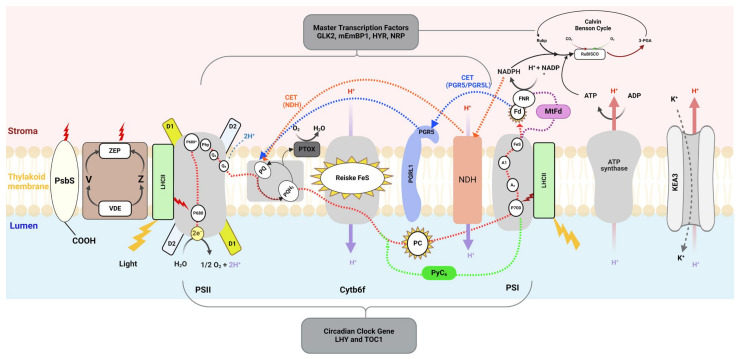
A comprehensive schematic diagram of the electron transport chain (ETC) showing linear and cyclic electron flow along with the strategies used to manipulate or enhance the electron-transfer process. The common photosynthetic machinery complex is shown by a grey box. The dotted red lines represent the linear electron transfer (LET). It starts with water oxidation, and the electron transfers through PSII-PQ-Cytb6f-PC-PSI. PSI then transfers the electrons to ferredoxin, which reduces NADP+ to NADPH. During LET, protons are translocated from the stroma to the lumen, generating a proton gradient across the thylakoid membrane. This proton gradient drives ATP synthesis from ADP by ATP synthase and protonates the light-harvesting complex, changing it from the light-harvesting mode to light-dissipative mode through the PsbS gene (light yellow oval box) and xanthophyll cycle (light brown square box). Proteins involved in the conventional electron-transfer chain for which overexpression/knockout has been shown to affect photosynthesis are enclosed in a star shape. The overexpression of the nuclear-encoded D1 gene discussed in the text is shown by the yellow box. Moreover, the exogenous overexpression of soluble electron transfer cytochrome c6 and ferredoxin gene from algal Porphyra yezoensis (Py) and Methanothermobacter thermautotrophicus (MtFd) are depicted in green and violet boxes, respectively. In parallel to LET, other alternative electron transfers are also present, such as cyclic electron transfer (CET). There are two types of CET; the dotted blue lines show PGR5/PGRL1-type CET, while the dotted orange lines show NDH-dependent CET. Additionally, the KEA3 potassium ion channel, which influences photosynthetic efficiency, is highlighted. The square boxes represent master regulators that control various genes involved in photosynthetic components. Another square box illustrates circadian clock genes that regulate the expression of photosynthesis-related genes, particularly those involved in pigment regulation. The ATP and NADPH generated during the light reactions of photosynthesis are utilized by the Calvin-Benson cycle (CBC) to fix carbon dioxide and synthesize carbohydrates.

One promising approach for optimizing the photosynthetic electron transport chain (PETC) is the reintroduction of the photosynthetic machinery components that were lost during evolutionary processes. This strategy aims to enhance photosynthetic efficiency by restoring the functionality of these ancestral elements, potentially leading to improved carbon fixation and overall plant productivity. Chida et al. (2007) provided the first evidence that gains in plant development can be driven by increases in electron transport [41]. They also demonstrated that the expression of cytochrome (Cyt) c6 from the algal *Porphyra yezoensis* (Py) in the chloroplasts of Arabidopsis resulted in increased starch and chlorophyll content, as well as ATP and NADPH. Additionally, the absorption of CO_2_, photosynthetic electron transport efficiency, and biomass increased in tandem with these alterations [41]. Chida et al. (2007) also showed that, in vivo, algal Cyt c6 can transport electrons from the Cyt b6f complex to Arabidopsis photosystem I (PSI) more quickly than the native plastocyanin of the plant (Figure 1). Similarly, Yadav et al. (2018) found that overexpressing Cyt c6 from *Ulva fasciata* (Uf) in tobacco had similar outcomes [42].

Recently, the heterologous expression of the ferredoxin gene from *Methanothermobacter thermautotrophicus* (MtFd) in rice significantly enhanced the electron transport rate (ETR), leading to increased grain weight, length, and width (see Figure 1). The increased electron flow, driven by MtFd overexpression, resulted in higher levels of NADPH [43]. However, in C3 plants like rice, the availability of CO_2_ is insufficient to fully utilize the excess NADPH for carbon fixation. This inadequate CO_2_ concentration leads to an accumulation of electrons, causing the production of reactive oxygen species (ROS) as excess electrons are diverted to oxygen. Consequently, the enhanced ETR improves agronomic traits but also elevates oxidative stress due to the imbalance between electron transport and CO_2_ assimilation [44]. Similarly, the overexpression of the Arabidopsis *AtPETE2* gene enhanced photosynthetic activity and improved seed yields in *Camelina sativa* [45]. Both the exogenous and endogenous overexpression of soluble electron transporters can boost plant growth. For instance, increasing the expression level of native plastocyanin and ferredoxin in plants showed enhanced electron transport, ultimately improving plant growth [46]. This demonstrates that it is not the evolutionary origin but the quantity of specific proteins that limits electron transport and affects plant growth. Enhancing the expression levels of these proteins, whether endogenous or exogenous, can significantly boost photosynthetic activity and growth.

Additional strategies to increase photosynthetic efficiency per leaf include augmenting the production of PETC machinery or extending the functional lifespan of photosynthetic machinery, such as promoting the stay-green phenotype. While these approaches can enhance the generation of ATP and NADPH, their effectiveness is contingent upon synchronization with downstream processes to fully utilize the supplied ATP and NADPH, referred to as sink capacity. If the sink capacity is not aligned with the increased production of ATP and NADPH, it may result in negative feedback, leading to elevated oxidative stress. Enhancing the generation of various components of the PETC machinery can be achieved through the targeted regulation of individual gene expression. For instance, the Cyt b_6_f complex plays a crucial role in photosynthetic electron transport. It mediates electron flow between PSII and PSI, oxidizes plastoquinol (PQH_2_), and reduces plastocyanin to provide ATP and NADPH for photosynthetic carbon fixation [47,48]. Eight distinct subunits make up this complex: two are encoded in the nucleus (PetC/Rieske FeS and PetM), while the other six are found in the chloroplast genome (PetA/Cyt f, PetB/Cyt b6, PetD, PetG, PetL, and PetN) [49,50]. Earlier research demonstrated that the levels of the Cyt b_6_f complex might be altered by decreasing the Rieske FeS protein accumulation [51]. The Cyt b_6_f complex is a crucial factor in determining the electron transport rate [52], as demonstrated by antisense experiments that suppress the Rieske FeS protein (PetC) and the use of Cyt b6f inhibitors [53]. Research using antisense techniques has shown that reducing Rieske FeS protein accumulation leads to a decline in photosynthetic electron transport, biomass, and seed output [51]. Plants with decreased levels of the Rieske FeS protein also had lower Chl a/b ratios, lower ATP synthase complex levels, and a smaller trans-thylakoid pH gradient [54]. 

In contrast, increasing the expression of the Rieske FeS protein could potentially enhance photosynthetic electron flow through the Cyt b6f complex, given that the Rieske FeS protein is one of the subunits required for the stable assembly of the Cyt b_6_f complex [53]. Overexpressing the Rieske FeS protein in Arabidopsis resulted in significant increases in CO_2_ assimilation and relative electron transport rates (see Figure 1). More significantly, it contributed to a 28–73% increase in biomass and a 52% increase in seed yield [55]. These results indicate that boosting electron transport might enhance photosynthesis and yields [56]. 

In another study, the nuclear expression of the chloroplast gene *psbA*, which encodes the D1 protein, driven by a heat-responsive promoter, significantly enhanced the amount of PSII subunits by protecting the degradation of D1 protein, ultimately improving photosynthesis efficiency (Figure 1). As a result, transgenic plants, including Arabidopsis, tobacco, and rice, showed increased biomass and grain yield under normal and heat-stress conditions [57]. However, the native *psbA* gene belongs to the plastid genome [58]. Therefore, this bioengineering strategy presents a promising outcome whereby plastid-encoded proteins can be effectively modified without relying on plastid transformation [57]. This is a crucial finding because plastid transformation is not a standard procedure and, so far, is only efficient in a few species. Since photosynthesis components are derived from chloroplast and nuclear genomes, engineering individual genes to increase the amount of photosynthesis subunits is a relatively complex process. Therefore, identifying and tuning a master regulator transcription factor to alter the expression of a set of genes regulating photosynthesis or involved in the synthesis of photosynthetic components is another approach.

Yu et al. (2014) pinpointed a set of identified transcription factors regulating the genes involved in photosynthesis in Arabidopsis by integrating genomic sequences, transcription factor binding information, and gene expression data [59]. Through this combined approach, the researchers identified 13 transcription factors related to photosynthesis, with some previously known and others newly identified as candidates. One of the identified transcription factors is maize (m) Embp-1. In a subsequent study, Perveen et al. (2021) demonstrated that the transgenic expression of mEmBP-1 in rice led to upregulating numerous photosynthesis-related genes [60]. This resulted in enhanced photosynthetic CO_2_ uptake, increased biomass, and higher grain yield. These findings highlight mEmBP-1 as a major regulator of photosynthesis, offering significant potential for improving crop yields by enhancing photosynthetic efficiency. Ambavaram et al. (2014) also identified a transcription factor named higher yield regulation (HYR) through a gene-regulatory network. The overexpression of HYR in rice resulted in increased grain yield and acted as a master regulator to express photosynthesis genes under different environmental conditions [61].

A wide range of genes involved in photosynthesis are controlled by Golden 2-like transcription factors (GLKs), which play a crucial role in enhancing plant growth and photosynthetic capacity, thereby significantly influencing chloroplast development and function. GLK not only expresses the nuclear-encoded genes related to chlorophyll biosynthesis, the light-harvesting complex, and soluble electron transporters but also regulates plastid-located genes for the photosystem reaction center [62]. Recent studies have highlighted the potential of transcription factors in enhancing photosynthesis and crop yield. It was reported that the overexpression of mGLK2 under the maize *Ubi* promoter significantly improved photosynthesis and grain yield [45]. More recently, Yeh et al. (2022) demonstrated that the increased expression of both maize GLK genes in rice, driven by their native promoters, resulted in higher grain yields [46].

Additionally, a transcription factor named Negative Regulator of Photosynthesis 1 (NRP1) was identified through the co-expression analysis of rice RNA sequencing data [47]. A significant negative correlation was observed between the expression of NRP1 and photosynthesis-related genes. In field conditions, the knockout lines of NRP1 exhibited decreased photosynthetic efficiency and biomass compared to wild-type plants, whereas the overexpression lines of NRP1 showed increased photosynthetic efficiency and biomass production. These findings underscore the critical role of NRP1 in regulating photosynthesis and plant growth in rice [47]. Thus, identifying and manipulating transcription factors that regulate photosynthesis-related genes could have a substantial impact on crop yields (Figure 1).

Extending the time span for efficient photosynthesis throughout a day or season can be achieved using various strategies, including promoting the stay-green phenotype or delayed senescence, optimizing the circadian rhythm, and manipulating the antenna size of the light-harvesting complex (LHC). Efforts to prolong active photosynthesis within a season have focused on developing crops exhibiting the stay-green or late senescence phenotype. Several genes have been identified for stay-green/late senescence phenotype, including non-yellowing NYL, Stay Green Rice 1 and 2 (SGR1 and SGR2), among others [63]. A recent genome-wide association study identified eight candidate genes related to the stay-green phenotype, with trehalose-6-phosphate synthase being one of the promising candidates [64]. Additionally, a more recent study indicated that reducing the activity of Aspartic Protease 1 (APP1) in wheat can extend photosynthesis periods, thus increasing photosynthetic rates, grain size, and yields. APP1 degrades PsbO, a component of photosystem II required for photosynthesis. Knockout mutants of APP1 showed increased yields and a stay-green phenotype, whereas overexpressing APP1 caused early senescence. Although the stay-green phenotype appears promising on paper, there are several notable drawbacks to this approach. For instance, the stay-green phenotype may be disadvantageous in drought-prone areas where an extended crop lifespan cannot be supported. Furthermore, this phenotype requires an adequate nitrogen supply to generate new cells, making its implementation more complex and expensive [65].

Furthermore, light intensity changes throughout the day, not just through transitions between shade and light but also due to the sun’s movement, leading to significant differences in light intensity from morning to night. Extending the period during which the active photosynthetic machinery remains active for a longer period within a day could substantially enhance light utilization and overall photosynthetic efficiency. A key factor in achieving this advantage is the circadian rhythm. Circadian rhythm, an intrinsic 24 h cycle, regulates gene expression by activating and repressing specific genes at different times of the day. Photosynthesis genes are also regulated by this rhythm [66,67,68]. The regulation of photosynthesis by the circadian clock is crucial, particularly during dawn and dusk, as it aligns plant physiological activities with the day-night cycle [69]. Recent research has identified the significant roles of the circadian clock components’ late elongated hypocotyl (LHY) and the timing of CAB expression 1 (TOC1) in controlling photosynthetic processes. In the native tobacco plant (*Nicotiana attenuata*), experiments that silenced the *NaLHY* and *NaTOC1* genes resulted in disrupted photosynthetic rhythms. These *NaLHY*-silenced plants displayed reduced growth rates and altered responses to light, including a premature reduction in photosynthetic efficiency at dusk and an unusual increase in response to light during the night, suggesting that LHY helps prevent unnecessary photosynthetic activity at night [69]. Additionally, studies on beans and cotton have shown that circadian rhythms influence the composition of photosynthetic pigments. Under constant-light conditions, the chlorophyll a/b ratio oscillates, peaking during subjective nights, indicating a regulatory mechanism that enhances light harvesting and reduces photodamage [70]. The relationship between circadian rhythms and photosynthesis is a critical area of research with significant implications for agricultural productivity. By understanding and manipulating the components of these rhythms, it may be possible to extend the duration of active photosynthesis within a day. This extension can potentially enhance photosynthetic efficiency and, consequently, boost crop yields (Figure 1).

Nanoparticles have shown significant promise in enhancing photosynthesis [71]. Nayeri et al. (2023) demonstrated that Ag/ZnO core-shell nanoparticles could improve photosynthesis and growth rates in plants. These nanoparticles enhance light-harvesting efficiency and improve the overall photosynthetic capacity by boosting photosynthesis [72]. A recent study explored the enhancement of photosynthetic efficiency in apple trees using nitrogen-doped carbon dots (N-CDs). These N-CDs were synthesized via a hydrothermal method, ensuring their particle sizes and heights allowed for effective penetration into plant cells. Apple trees were sprayed with N-CD solutions in the field, and various physiological indices, including the net photosynthetic rate (Pn) values of leaves and fruit quality metrics, were measured. It was found that light-excited N-CDs could transfer electrons to plastoquinone (PQ), resulting in an increased production of reduced PQH_2_. This electron-transfer process was confirmed through high-performance liquid chromatography (HPLC) and various fluorescence measurements, indicating that N-CDs could effectively complement the PETC. This led to increased photosynthetic rates and better plant growth, significantly improving apple fruit quality [73]. These findings provide a basis for designing more efficient nanomaterials, offering a promising approach to enhance photosynthesis across various plant species. However, despite their potential benefits, the environmental effects and behavior of engineered nanoparticles remain largely unknown, necessitating careful consideration before their use in agro-food systems.

### 2.2. Modification of Calvin-Benson Cycle

**Figure 2:**
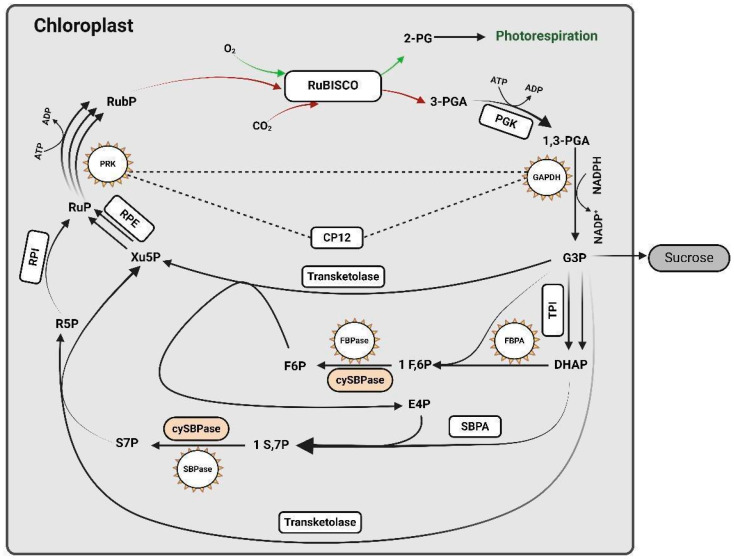
A systematic representation of the Calvin-Benson Cycle. The eleven enzymes of the CBC are indicated in white boxes with black borders. Endogenous enzymes that have been genetically manipulated in previous studies, as discussed in our review, are enclosed in star shapes. The formation of the GAPDH-CP12-PRK complex is depicted by dotted lines. The exogenous overexpression of cyanobacterial (cy) genes is shown in light brown boxes. Enzymes: RubisCO, ribulose-1,5-bisphosphate carboxylase/oxygenase; PGK, phosphoglycerate kinase; GAPDH, glyceraldehyde-3-phosphate dehydrogenase; TPI, triose phosphate isomerase; FBPA, fructose-1,6-bisphosphate aldolase; FBPase, fructose-1,6-bisphosphatase; transketolase; SBPA, sedohuptulose bisphosphate aldolase; SBPase, sedoheptulose-1,7-bisphosphatase; RPE, ribulose-5-phosphate 3-epimerase; RPI, ribose-5-phosphate isomerase; PRK, phosphoribulokinase. Metabolites: RuBP, ribulose-1,5-bisphosphate; 3-PGA, 3-phosphoglycerate; 1,3-PGA, 1,3-bisphosphoglycerate; G3P, glyceraldehyde-3-phosphate; DHAP, dihydroxyacetone phosphate; 1F,6P, fructose-1,6-bisphosphate; F6P, fructose-6-phosphate; Xu5P, xylulose-5-phosphate; E4P, erythrose-4-phosphate; 1S,7P, sedoheptulose-1,7-bisphosphate; S7P, sedoheptulose-7-phosphate; RuP, ribulose-5-phosphate.

The Calvin-Benson cycle (CBC) is essential for photosynthesis, converting atmospheric CO_2_ into the organic molecules vital for plant growth. This cycle, discovered over 70 years ago, is conserved across all plant species, from cyanobacteria to higher terrestrial plants. It comprises eleven enzymes and proceeds through three stages: carboxylation, reduction, and ribulose-1,5-bisphosphate (RuBP) regeneration [74] (Figure 2). Understanding and manipulating these enzymes via genetic modification can enhance photosynthetic efficiency and crop yields.

In the carboxylation phase, ribulose-1,5-bisphosphate carboxylase/oxygenase (RubisCO) catalyzes the reaction of CO_2_ with RuBP to produce 3-phosphoglycerate (PGA). This reaction is the limiting step in carbon fixation under saturating light and limited CO_2_ concentration because, under these conditions, RubisCO activity is rate-limiting [75]. RubisCO also reacts with oxygen, producing 2-phosphoglycolate (PG) and competing with carbon fixation [76]. Therefore, significant efforts have been made to engineer RubisCO to achieve a high carbon-fixation efficiency. For the latest updates on RubisCO engineering to enhance photosynthesis, recent reviews provide comprehensive insights [77,78,79]. There is a shift in the equilibrium of the CBC as CO_2_ concentrations increase and light intensity decreases, affecting RuBP synthesis [75]. This shift also affects the reductive and regenerative phases. Here, we explain how these reductive and regenerative phases are manipulated by using genetic modification to achieve enhanced photosynthesis and yield.

In recent years, much of the research has been focused on improving the CBC through the overexpression of key enzymes involved in the cycle. Initially, efforts centered on the endogenous overexpression of single CBC enzymes. One notable enzyme is sedoheptulose-1,7-bisphosphatase (SBPase), essential for RuBP regeneration. The ectopic expression of SBPase in various plant species, including tobacco, rice, and Arabidopsis, has resulted in significant increases in photosynthetic rates and biomass [55,80]. In tobacco, overexpressing SBPase resulted in a 30% greater leaf fresh weight than controls [81] (Figure 2). Similar results were seen in wheat by upregulating SBPase activity [82].

Other enzymes, such as fructose-1,6-bisphosphatase (FBPase) and fructose-1,6-bisphosphate aldolase (FBPA), have been studied to determine their roles in photosynthesis. The overexpression of FBPase improved photosynthesis and growth in tobacco, while reducing FBPase activity decreased photosynthetic rates [83,84] (Figure 2). Uematsu et al. showed that overexpressing FBPA in tobacco under high CO_2_ (700 ppm) increased photosynthetic CO_2_ fixation by 1.5-fold, aldolase activity by 1.4–1.9-fold, and biomass by 70-120%. However, the effects were less pronounced under ambient CO_2_ conditions [85]. Expressing cyanobacterial enzymes like FBPase (cyFBPase) and SBPase (cySBPase) in higher plants confirmed the benefits observed with plant enzymes. Tamoi et al. (2006) reported that cySBPase-expressing tobacco plants had increased growth and photosynthetic CO_2_ fixation, resulting in a 1.5-fold increase in biomass (Figure 2).

The CBC operates efficiently under light conditions but downregulates during darkness to conserve energy. Glyceraldehyde-3-phosphate dehydrogenase (GAPDH) and phosphoribulokinase (PRK) are critical in this regulation. GAPDH reduces 1,3-bisphosphoglycerate to glyceraldehyde-3-phosphate (G3P). Overexpressing GAPDH in rice, alongside RubisCO, improved photosynthesis and biomass production [86]. PRK is vital for RuBP regeneration; increased PRK expression boosts RuBP production but causes metabolic imbalances if not regulated [87]. GAPDH and PRK form complexes with CP12, a regulatory protein, in low light, inactivating these enzymes. In light, thioredoxins activate CBC enzymes by reducing disulfide bonds in GAPDH and PRK, dissociating the CP12-GAPDH-PRK complex and reactivating the enzymes [88]. This regulation prevents photoinhibition or oxidative stress and optimizes the CBC for varying light conditions [89].

Computational models have identified potential targets for genetic modification, suggesting that a multi-target approach, involving the simultaneous manipulation of several CBC enzymes, may be more effective than targeting individual enzymes [90]. Co-overexpressing SBPase and FBPA in tobacco increased biomass and photosynthetic efficiency [81].

However, overexpressing CBC enzymes does not always enhance photosynthesis. For example, overexpressing transketolase in tobacco resulted in negative growth and reduced photosynthetic rates, indicating metabolic imbalance [91,92]. Thus, a balanced approach is crucial to avoid disrupting the CBC’s complex network of reactions.

In conclusion, the CBC is an integrated system where enzyme activities are interdependent. Manipulating CBC enzymes can boost photosynthesis and crop yields but requires understanding the regulatory networks and metabolic fluxes within the cycle. Increasing the activity of one enzyme without considering system-level constraints can lead to suboptimal outcomes. Continued research, integrating experimental and computational approaches, is essential for realizing these strategies’ potential in agriculture. A multi-target approach, combining overexpression, downregulation, and the dynamic regulation of multiple enzymes, may be the most effective strategy for enhancing photosynthetic efficiency and crop yields (Figure 2).

### 2.3. Modification of Photorespiratory Process

**Figure 3:**
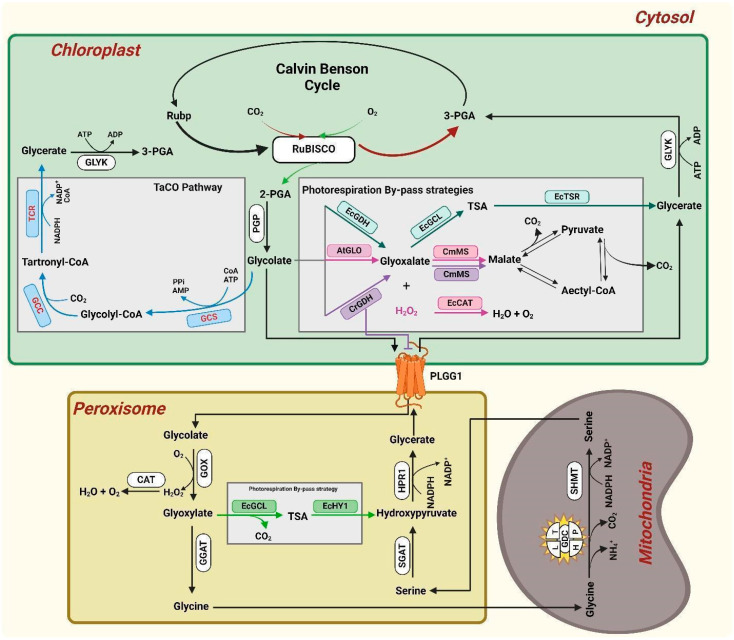
This diagram illustrates the natural photorespiration process alongside four engineered by-pass systems and a novel enzymatic strategy to enhance photosynthesis efficiency. The natural photorespiration pathway, depicted with black arrows, involves a series of reactions across the chloroplast, peroxisome, and mitochondria, with key enzymes in white boxes (cytosolic reactions are not presented here for simplicity). Proteins involved in conventional photorespiration, whose overexpression affects photosynthesis, are enclosed in star shapes. Engineered pathways designed to reduce natural inefficiencies are enclosed in grey boxes, each color-coded to represent different bypass strategies. These include modifications within the chloroplast and peroxisome to reduce the loss of carbon and energy typically associated with photorespiration. A new-to-nature enzymatic approach (Tartronyl pathway, TaCo) is also highlighted. In the TaCo pathway, glycolate is first converted into glycolyl-CoA by the enzyme GCS. The enzyme GCC carboxylates glycolyl-CoA to form tatronyl-CoA. Finally, tatronyl-CoA is transformed into glycerate by the enzyme TCR, showcasing the potential to further optimize carbon fixation through synthetic biology. This comprehensive representation integrates both traditional and innovative tactics, providing a multi-faceted view of current advancements in photorespiration modification. The details for each engineered strategy highlighted here are explained in the text. Species from which genes are sourced to engineer by-pass processes include the following: At, *Arabidopsis thaliana*; Cm, *Cucurbita maxim*; Cr, *Chlamydomonas reinhardtii*; Ec, *Escherichia coli*. Enzyme names: CAT, catalase; GCC, glycolyl-CoA carboxylase; GCL, glyoxylate carboligase; GCS, glycolyl CoA synthase; GDC, glycine decarboxylase; GDH, glycolate dehydrogenase; GGAT, glutamate glyoxylate aminotransferase; GLYK, glycerate kinase; GLO, glycolate oxidase; GOX, glycolate oxidase; HPR1, hydroxypyruvate reductase 1; HY1, hydroxypyruvate isomerase; MS, malate synthase; PGP, phosphoglycolate phosphatase; RubisCO, ribulose-1,5-bisphosphate carboxylase/oxygenase; SGAT, serine glyoxylate aminotransferase; SHMT, serine hydroxymethyl transferase; TSR, tartronic semialdehyde reductase. Metabolite: 2-PG, 2-phosphoglycolate; 3-PGA, 3-phosphoglycerate; RuBP, ribulose-1,5-bisphosphate; TSA, tartronic semialdehyde; PLGG1, plastidial glycolate/glycerate transporter 1 (the transporter is shown by orange helix-like structure).

Photorespiration is a major bottleneck in photosynthesis. This process consumes a significant amount of energy (around six reducing equivalents and eight ATPs) to convert two molecules of PG into one molecule of PGA. This process spans three subcellular compartments (chloroplast, peroxisome, and mitochondria) and involves several enzymes (Figure 3). At 25 °C and under current atmospheric CO_2_ conditions, approximately 30% of the CO_2_ previously fixed during photosynthesis is lost/released in the mitochondria due to photorespiration, and this loss is exacerbated with the increasing temperature. Moreover, this mechanism produces one molecule of ammonia (NH_3_) and hydrogen peroxide (H_2_O_2_) for each of the two oxygenation events required for the synthesis of glycine [29,93]. According to Rojas et al. (2012), H_2_O_2_ and NH_3_ play crucial roles in plant fitness, including disease resistance and nitrogen assimilation [94]. However, their buildup to toxic levels can be hazardous [95]. Additionally, re-assimilating NH_3_ through the actions of glutamine synthetase and glutamine 2-oxoglutarate aminotransferase raises the energetic costs [96] (Figure 3). All these factors make photorespiration a focal point for improving photosynthesis efficiency. One prevalent approach to mitigate the negative impacts of photorespiration involves modifying the endogenous proteins that play roles in natural photorespiration.

Modeling studies have shown that a slight decrease in the amounts of photorespiratory proteins may improve nitrogen distribution, which could increase CO_2_ uptake [97]. However, earlier research has demonstrated that decreasing the flux along the photorespiratory route in high-photorespiration environments, such as those with high temperatures, results in a drop in photosynthetic efficiency [98]. For instance, the knockdown of the GDC (glycine decarboxylase complex) H-protein in rice and P-protein in potatoes reduces growth rates and photosynthesis, the flux through the photorespiratory cycle, as well as the rate at which mitochondria oxidize glycine [99,100]. These findings align with past research by Wingler et al. (1997) [101], who discovered a heterozygous barley mutant exhibiting a 50% decrease in GDC H-protein levels. Since a decrease in the activity of the photorespiratory pathway’s enzymes had adverse consequences, researchers have investigated whether overexpressing GDC components may improve photorespiration flow and lessen the buildup of photorespiratory intermediates. The overexpression of the H- or L-protein in Arabidopsis enhanced photosynthesis and increased crop yield and productivity [55,102]. Additionally, recent studies on tobacco have demonstrated that the mesophyll-specific overexpression of the H-protein leads to improved growth and greater biomass (up to 48%) when cultivated in field and greenhouses [103]. On the other hand, T-protein overexpression in Arabidopsis did not change photosynthetic CO_2_ absorption or enhance plant growth [102,104]. Surprisingly, increased sucrose (and fructose and maltose) levels were also observed with the overexpression of the L-protein [102,104,105]. Similarly, the overexpression of the L-protein results in an improved capacity for photorespiratory metabolism, which modifies the flow of carbon through the tricarboxylic acid cycle (TCA) [105] (Figure 3). 

Earlier studies demonstrated that 2PG is detrimental as it inhibits two CBC enzymes, specifically, PRK in spinach [106] and triose-phosphate isomerase (TPI) in peas [107], both of which are required for the CBC and starch synthesis. Recent research supports this theory, showing that 2PG also inhibits TPI and SBPase, slowing Arabidopsis’ ability to synthesize starch [108]. Further research has demonstrated that glyoxylate inhibits RubisCO activation in isolated chloroplasts. The probability of CBC inhibition is decreased when there is a decrease in the levels of this photorespiratory metabolism due to the increased stimulation of GCS (glycine cleavage system) activity [53,107,109]. Reducing photorespiration via lowering the activity of GCS enzymes has largely been abandoned, given the outcomes of both the down- and up-regulation of proteins in the photorespiratory pathway. Therefore, a promising approach to enhance photosynthesis efficiency involves creating a photorespiration bypass that targets the metabolism of 2PG. This strategy ensures the release of CO_2_ near RubisCO within the chloroplast or peroxisome rather than in the mitochondria, optimizing the conditions for carbon fixation [93,110,111,112] (Figure 3).

Several photorespiration-bypass strategies have been developed, as reviewed by Jin et al. (2023) [77]. One such bypass was pioneered by Kebeish et al. (2007) [113], who integrated five genes from the *Escherichia coli* (*E. coli*) glycolate pathway into Arabidopsis, allowing chloroplastic glycolate to be directly converted to glycerate within chloroplasts (see Figure 3). This genetic modification created a bypass that avoided the energy-intensive steps involving the cytosol, peroxisomes, and mitochondria, thus reducing the energy required for the typical recycling of glycolate to glycerate in the natural C3 photorespiration process, which ultimately enhances photosynthesis and biomass [113]. This enhanced photosynthesis is attributed to the increased CO_2_ concentration around RubisCO and reduced ATP consumption by avoiding ammonium re-fixation. The same pathway has also been successfully implemented in various other plant species to evaluate its applicability across a diverse array of species [114,115]. 

Similarly, the second bypass was generated by introducing two enzymes from *E. coli* into the peroxisomes of tobacco: glyoxylate carboligase (EcGCL) and hydroxypyruvate isomerase (EcHYI). This pathway allows glyoxylate to be directly converted to hydroxypyruvate within the peroxisome. Specifically, EcGCL first converts glyoxylate into tartronic semialdehyde (TSA), which is then isomerized into hydroxypyruvate by EcHYI (Figure 3). This bypass competes with the natural conversion of glyoxylate to glycine via glutamate glyoxylate aminotransferase (GGAT), thereby avoiding the production of excessive ammonia in the mitochondria [116]. 

Maier et al. (2012) [111] explored another engineered pathway in Arabidopsis chloroplasts for glycolate metabolism to enhance photosynthetic efficiency. They introduced a combination of endogenous and exogenous enzymes, including the relocation of glycolate oxidase from Arabidopsis (AtGLO) from the peroxisome to the chloroplast, catalase from *E. coli* (EcCAT), and malate synthase from *Cucurbita maxima* (CmMS). This pathway enabled the complete catabolism of glycolate to CO_2_, utilizing both malic enzyme and pyruvate dehydrogenase (already present in the chloroplast) to facilitate the process (Figure 3). While this approach showed promise in increasing biomass and photosynthesis, particularly under low-light and short-day conditions, its applicability in light-loving plants like rice may be limited due to the environmental specificity. Building on this, South et al. (2019) made a significant advancement in photorespiration bypass by integrating the approaches of Kebeish and Maier in tobacco chloroplasts, along with a clever modification of silencing the plastidial glycerate/glycolate transferase (PGLL1) protein to avoid the leakage of glycolate into the peroxisome [117]. This enabled the complete conversion of glycolate into CO_2_ within the chloroplast. Additionally, they used the GDH enzyme from *C. reinhardtii* (CrGDH) instead of using AtGLO to restrict H_2_O_2_ formation (Figure 3). This strategy not only conserved energy but also enhanced the CO_2_ concentration around RubisCO, leading to a marked increase in biomass under field conditions [117].

Engineering naturally existing enzymes and pathways has been the most conventional approach to improving plants’ carbon capture [113,117]. However, the rise of artificial intelligence and state-of-the-art synthetic biology techniques allows scientists to creatively redesign plant metabolism beyond natural evolutionary constraints [118]. In addition to potentially extending the biological solution space, such engineering approaches can offer innovative new-to-nature pathways that outperform their natural ones. Several of these new-to-nature solutions have been successfully tested in vitro and are awaiting in vivo transplantation [119,120]. The most successful example of this is the introduction of the Tartronyl Co-A (TaCO) pathway. The TaCo pathway, developed by Trudeau et al. (2019) [121] and further refined by Scheffen et al. (2021) [119], offers a novel method to enhance photosynthetic efficiency by turning photorespiration from a carbon-releasing process into a carbon-capturing one. This cutting-edge pathway was designed using computationally guided pathway engineering, along with advanced enzyme engineering techniques, including microfluidics-based high-throughput screening. The TaCo pathway incorporates three new-to-nature enzymes, including a novel CO_2_-fixing enzyme, glycolyl-CoA carboxylase (GCC). This enzyme, initially designed through rational design and subsequently enhanced via random mutagenesis, dramatically increases its catalytic efficiency compared to natural CO_2_-fixing enzymes. By effectively capturing CO_2_ in the chloroplast, photorespiration normally releases CO_2_ (see Figure 3), and the TaCo pathway transforms a critical photosynthetic limitation into a significant gain. This could potentially increase biomass production irrespective of RubisCO activity [119]. The TaCo pathway exemplifies the power of innovative biotechnological advancements and holds promise for significantly improving agricultural productivity (Figure 3). 

### 2.4. Modification of Non-Photochemical Quenching

Photosynthesis is regulated by light, with the rate of photosynthesis responsive to increasing light intensity. However, photosynthetic organisms have an optimal level of light intensity for photosynthesis. Beyond this level, further increases in light intensity, known as excess light, have no additional effect on the photosynthetic rate and pose potential risks to plants. Excess excitation energy can lead to increased levels of reactive oxygen species (ROS), such as singlet oxygen ^1^O_2_, superoxide radical (O^−2.^), and hydrogen peroxide (H_2_O_2_) [122,123]. These ROS particularly damage photosystems, especially photosystem II (PSII) [124]. Plants have evolved natural photoprotective mechanisms to cope with excess light. These mechanisms act as safety valves to avoid or dissipate excess light before producing ROS that can damage reaction centers [123]. Photoprotection works through physiological means, such as leaf and chloroplast movement, or molecular mechanisms, such as dissipating excess light as heat through the non-photochemical quenching process (NPQ) and activating ROS-scavenging systems [125,126,127,128,129]. If plants cannot avoid excess light through these processes and ROS damage PSII, especially the D1 protein, the last line of defense is the repair cycle of D1 [130,131]. Here, we mainly focus on NPQ. 

In the field, plants experience continuous fluctuating light, and, often, the light intensity exceeds their optimal photosynthesis capacity. This variability is due to factors such as the sun’s passage across the day, shading from the neighboring plants, or sunlight intermittently penetrating through gaps in the leaves, creating brief high-light conditions known as sunflecks [132]. To cope with this, plants have adopted various strategies, including NPQ. NPQ is a complex dynamic phenomenon constantly changing in response to environmental constraints. Although NPQ is a photoprotective mechanism, it often competes with photosynthesis [133,134]. It has been theoretically shown that a fast recovery of the photosynthetic machinery from a photoprotective state to an un-photoprotective state can help improve canopy photosynthetic efficiency [8]. Later on, Kromdijk and his colleagues provided experimental evidence for this hypothesis [9].

NPQ has been investigated in detail, and, based on its relaxation kinetics in the dark, it has been divided into different components. The quickest and most efficient response against excess light energy is called energy-dependent quenching (qE) [135,136,137]. The moderate form of quenching for several minutes is Zeaxanthin-dependent quenching (qZ) [138]. The slowest components of NPQ include photoprotective quenching (qH) and photoinhibitory quenching (qI), which take hours to relax [139,140,141].

According to the literature, qE is regulated through a pH gradient built across the thylakoid membrane (ΔpH). When light is absorbed by the leaf, electrons start to move from PSII to PSI. During this process, protons (H^+^) are transported from the stroma to the lumen of the thylakoid, leading to subsequent ATP synthesis (see Figure 1). This increase in the H^+^ concentration on the lumen side is proportional to the light absorbed by the antenna complex. The excess absorbed light results in the saturation of the CBC and the accumulation of ATP, which limits the consumption of ADP and Pi, thus decreasing the ATPase activity. Since ATPase acts as a modulator on the balance between the proton concentrations on the two sides of the thylakoid membrane, decreasing ATPase activity subsequently hinders the return of protons to the stroma side. As a result, the lumen side becomes acidic, and a proton gradient builds across the thylakoid membrane [122]. 

Cyclic electron flow (CEF) also plays a key role in building the pH gradients across the thylakoid membrane. CEF around PSI generates ATP without accumulating NADPH in chloroplasts. CEF around PSI is also identified as a photoprotection mechanism, especially protecting PSI from photodamage [142]. There are two main categories of CEF: Fd-quinone oxidoreductase (FQR-dependent CEF) [143] and NAD(P)H dehydrogenase (NDH-dependent CEF) pathways [144,145] (refer to Supplementary Figure S2 for a detailed illustration of classic LEF and CEF pathways, providing additional clarity). In the former, the electron is derived from the PSI to Fd present at the acceptor side of PSI and then reverts to the plastoquinone pool through FQR. This pathway involves two genes: one is the proton gradient regulation 5 (PGR5) gene, and one is the proton gradient regulation-like 1 (PGRL1) gene [143] (see Figure 1 and, for the sake of clarity, we also provide Supplementary Figure S2, which shows typical LET and CET). FQR-dependent CEF mainly contributes to ΔpH. Arabidopsis mutants for the PGR5 and PGRL1 genes, which regulate CEF around PSI, show that CEF increases the transport of H^+^ from the stroma to the lumen, subsequently decreasing the pH level on the lumen side. Arabidopsis mutants lacking these genes show lower NPQ levels than their wild-type counterparts [143]. 

Ion-transport channels, particularly the KEA3 antiporter, play a critical role in regulating the proton gradient across the thylakoid membrane, essential for photosynthetic efficiency [146]. The KEA3 antiporter mediates the exchange of protons from the lumen with potassium ions from the stroma, thus decreasing the lumenal proton concentration and facilitating the rapid relaxation of qE-dependent NPQ (see Figure 1). KEA3 is regulated by light intensity and the chloroplast energy status. The C-terminal domain of KEA3, exposed to the stroma, senses changes in pH and nucleotide levels, such as ATP and ADP, triggering conformational changes in KEA3 and modulating its activity to optimize photoprotection [147]. During transitions from high to low light, KEA3 becomes active, exporting protons and decreasing the proton gradient, which, in turn, partly suppresses zeaxanthin accumulation. This accelerates the response of photosynthesis to low-light periods by reducing qE-dependent NPQ dissipation. Under excess light conditions, where high qE is necessary for photoprotection, KEA3 remains inactive [148]. Furthermore, Wang et al. (2016) identified a mutant allele of KEA3, known as disturbed proton gradient regulation (dpgr), which exhibited reduced NPQ in artificial air conditions (CO_2_-free with low O2). This phenotype was further pronounced in mutant backgrounds affecting PSI cyclic electron transport (double mutant; pgr5 and dpgr) [149]. This regulation ensures a dynamic response to fluctuating light conditions, enhancing photoprotection and overall photosynthetic efficiency.

The ΔpH triggers qE-type NPQ either directly or indirectly. When the H^+^ concentration increases on the lumen side, qE is directly triggered by the protonation of the acidic residues of specific proteins (LHCSR in algae and PsbS and LHCs in higher plants) that are exposed to the lumen (see Figure 1). This protonation leads to the conversion of the antenna system from light-harvesting mode to light-dissipative mode. In addition, the VDE enzyme also activates when the pH of the thylakoid decreases to 5.5, converting violaxanthin to zeaxanthin, which also plays a role in regulating qE [150] (Figure 1). However, PsbS is the most critical and well-studied gene regulating the NPQ process. This gene is completely knocked out in an Arabidopsis mutant line (npq4-1), which shows very low levels of NPQ, especially a decrease in the qE component. Despite this, the photosynthesis and xanthophyll cycle remain active in this mutant line. Furthermore, overexpressed lines of PsbS showed a high activity of qE. PsbS senses lumen acidification and exhibits a rapid response in the induction and relaxation of qE-type NPQ [151,152]. 

The composition of plant carotenoids is highly influenced by changing environmental conditions, especially the three carotenoids, violaxanthin, antheraxanthin, and zeaxanthin, which are highly susceptible to changing light environments. Their interconversion is known as the xanthophyll cycle. Under dark or low light conditions, only violaxanthin is present. When light intensity increases, violaxanthin first converts into antheraxanthin through mono-de-epoxidation and then further de-epoxidation converts into zeaxanthin via the violaxanthin de-epoxidase (VDE) enzyme, which is located on the lumen side (see Figure 1). The electron for the de-epoxidation process is donated by ascorbate [153]. Under low light, VDE in the lumen is present in a monomeric and inactive form. When the lumen becomes acidified upon exposure to excess light, VDE is activated through acidification; it dimerizes and binds to the thylakoid membrane where it works as a lipid-soluble substrate [154]. The higher level of zeaxanthin accumulation during excess light corresponds to higher NPQ. In contrast, the zeaxanthin epoxidase enzyme is present on the stromal side of the thylakoid membrane, and, upon returning from high light to low light, zeaxanthin is converted back into violaxanthin through epoxidation using zeaxanthin epoxidase (see Figure 1). This cycle is known as the xanthophyll cycle or V cycle [153]. This cycle plays a crucial role in regulating excess light, especially under fluctuating light conditions. It acts as an active photoprotection mechanism and balances the rate of the CBC through the rapid conversion of violaxanthin to zeaxanthin and vice versa [155]. However, compared to qE, zeaxanthin-dependent quenching is a bit slower. The complete conversion of zeaxanthin into violaxanthin under dark or low light conditions is a slow process, taking almost an hour, as shown in laboratory experiments [138,156]. This slow NPQ component, due to the accumulation of zeaxanthin, is found in higher plants and is known as zeaxanthin-dependent quenching (qZ). Dall’Osto et al. (2015) describe that qZ might be due to zeaxanthin binding to monomeric LHC, with CP26 (a component of LHC) being important for setting up this slow NPQ component. This slow NPQ component is not dependent on or linked with PsbS or ΔpH [156]. The Arabidopsis VDE-defective mutant (npq1 mutant), which lacks the formation of zeaxanthin under high light, shows similar NPQ induction for the first few seconds as its wild-type counterpart. However, it is notable that, after the initial NPQ induction rise, NPQ in the wild type continues to increase with prolonged light exposure, while no further rise is observed for the mutant lines. This suggests that the xanthophyll cycle is not effective for the initial NPQ induction, but it is necessary for achieving maximum NPQ production [157]. Additionally, the npq2 mutant, which accumulates zeaxanthin by preferentially blocking the zeaxanthin epoxidase, exhibited faster qE. This provides evidence that zeaxanthin formation is vital for complete qE activation [152].

In light of the above discussion and the hint from a theoretical study [8], a transgenic approach has been adopted to enhance the relaxation kinetics of NPQ by overexpressing the key NPQ regulators violaxanthin de-epoxidase (VDE), PsbS, and zeaxanthin epoxidase (ZEP) collectively known as the VPZ approach. This methodology has demonstrated promising results, as evidenced by a 15% increase in biomass in *Nicotiana tabacum* [9]. Furthermore, implementing the VPZ approach in soybeans resulted in a 20% boost in seed yield under field conditions [158]. In contrast, the VPZ approach did not lead to an increase in biomass in Arabidopsis and *Solanum tuberosum* [159,160]. This discrepancy might be attributed to the different stoichiometries of these proteins across various plant species, as suggested by modeling studies [161]. Therefore, to ensure the success of the VPZ approach, it is crucial to optimize the expression of the VPZ construct according to the native qE and qZ capacities of the selected species. Special attention should be given to tailoring the expression levels of these regulators to align with the specific physiological and biochemical contexts of the target plant species.

Similar to the violaxanthin xanthophyll cycle, there is another carotenoid cycle simultaneously working in higher plants called the lutein xanthophyll cycle. Like the violaxanthin cycle, it is also regulated by ΔpH and modulates the qE component of NPQ, specifically through lutein-related thermal dissipation. In this cycle, lutein epoxide (Lx) is converted to lutein (L) through de-epoxidation upon exposure to light. Conversely, under dark conditions, epoxidation converts L back to Lx using similar enzymes involved in the violaxanthin cycle. These two xanthophyll cycles operate in parallel; however, the violaxanthin cycle is more dominant and plays a critical role in higher plants under fluctuating light conditions [162]. In contrast to the ubiquitously present violaxanthin cycle, the Lx cycle is limited to plants with dense vegetation, usually forest species [162,163].

qH and qI are the slowest components of NPQ, but qH is completely different from qI. qH modulates a sustained form of energy dissipation that occurs in the trimeric light-harvesting complex II (LHCII). Its activation requires the plastid lipocalin (LCNP), whereas the protein suppressor of quenching 1 (SOQ1) inhibits LCNP, preventing uncontrolled heat dissipation [164,165]. A short-chain dehydrogenase reductase (ROQH1) is involved in the relaxation of qH. Strikingly, qH-deficient plants bleach under excess light, while the constitutive activation of qH causes severe light limitation and stunted growth. Plants unable to perform qH suffer from increased lipid peroxidation under stress [166]. Therefore, the proper regulation of qH is crucial for maintaining photosynthetic efficiency and preventing photodamage.

The photo inhibitory quenching (qI) is the slowest component of NPQ and is attributed to a decrease in the number of active reaction centers of PSII as a result of photodamage. The protein named D1 is highly susceptible to light damage [167]. Since qI is associated with the inactivation of D1 protein, it requires re-synthesis of the D1 protein for relaxation [15]. There is an efficient repair mechanism that continuously repairs the photodamaged D1 protein. The photoinhibition of PSII occurs only when the rate of damage exceeds the rate of repair. Therefore, qI influences photosynthesis only under high-light conditions or prolonged exposure to high light and is ineffective in low light. Since the relaxation process is based on the repair mechanism of the D1 protein, which is a slow process, qI needs hours to relax. By overexpressing the gene regulating D1 synthesis, the biomass increased [57]. A list of the major genes being targeted for the modification of various photosynthetic pathways is shown in Table 1.

### 2.5. Synergistic Enhancements in Photosynthesis: Multigene Modifications’ Impact

A combination of multigene modifications can improve photosynthesis. In other words, genes regulating carbon fixation, electron transport, and light capture can be modified simultaneously to enhance these processes, resulting in more efficient photosynthesis. This implies that plants might convert more sunlight into chemical energy more effectively, leading to faster growth and higher yields. Increased SBPase and FBPA activity in transgenic tobacco has been demonstrated to boost carbon absorption and biomass output [168]. Simkin et al. (2015) showed that overexpressing both FBPA and SBPase simultaneously in tobacco led to a cumulative biomass gain of 63% compared to 35% for SBPase alone [81]. Significantly higher photosynthetic rates at limiting CO_2_ levels have been observed due to the transformation of ictB into tobacco and Arabidopsis [169]. A similar behavior was seen in Arabidopsis plants expressing ictB, a putative inorganic carbon transporter, from Anabaena sp., providing more evidence that ictB might dramatically change carbon absorption rates [169]. According to Lieman-Hurwitz et al. (2003) [169], there is a possibility that ictB promotes photosynthesis and development in transgenic Arabidopsis plants by increasing the internal CO_2_ concentration around RubisCO, which eventually leads to a rise in enzyme activity. Gong et al. (2015) found that, while the expression of ictB in rice increased mesophyll conductance and photosynthetic carbon absorption by 18%, no discernible improvements in biomass, grain number, or grain weight were noted [170]. However, it has been demonstrated that ictB expression in *Glycine max* greatly increases photosynthetic CO_2_ uptake in both greenhouse and outdoor experiments. Under dry conditions, these plants also increased biomass output [171]. Similarly, rice plants expressing both ictB and the bifunctional cyFBP/SBPase were compared to plants expressing cyFBP/SBPase or ictB alone. The combined expression of the two proteins increased photosynthetic rates, tiller number, grain quantity, and grain weight [170].

After observing that increasing photorespiration in photosynthetic tissue positively impacted plant growth in both Arabidopsis and tobacco, Simkin et al. (2017) investigated the possibility that increasing photorespiration by overexpressing the GCS H-protein and simultaneously increasing the activity of two CBC enzymes (SBPase and FBPA) could have a cumulative effect on photosynthetic efficiency and yield [55]. Plants that expressed GCS H-protein, FBPA, and SBPase alone or in combination were assessed in this study. According to Simkin et al. (2017), controlling photorespiration and the CBC simultaneously increases yield output in both high- and low-light conditions. Interestingly, altering the photorespiratory pathway alone increased biomass output while showing no discernible rise in seed yield in these plants.

On the other hand, data show that plants overexpressing CBC enzymes exhibited an increase in biomass and seed output of approximately 22–38%. Simkin et al. (2017) found that altering the CBC and photorespiratory pathways simultaneously led to a combined increase in the seed output of approximately 63% compared to plants that overexpressed CBC enzymes alone. Since the plants were cultivated in a randomized grouping under identical conditions, the causes of these disparate impacts on seed output remain unexplained. However, variations in starch and sucrose levels in GCS H-protein-overexpressing lines have been linked to variations in carbon source/sink allocation [55]. These findings emphasize the necessity of assessing separate and multitargeted modifications in various plant species to pinpoint the precise targets and raise agricultural yields. 

## 3. Post-Translational Modification of Photosynthetic Machinery

Protein post-translational modifications (PTMs) are crucial components of the plant-regulation toolkit that controls carbon metabolism by allowing quick and frequently reversible changes to the characteristics of target proteins. Nature has provided PTMs as a means of transmitting perceived environmental and developmental cues. PTMs increase the complexity of the proteome, enabling metabolism in response to parallel inputs, thereby producing strong reactions and effective multi-signal integration. Additionally, the number of proteome combinations to accommodate fine-tuned responses grows considerably since distinct PTMs might target the same protein, occasionally the same amino acid residue, or have numerous inputs in different components of signaling pathways [172]. Determining the extent of plants’ control of carbon uptake and utilization is challenging. However, precisely controlling metabolic carbon fluxes is a desirable way to increase crop output. Most PTMs are absent from the specified target proteins, indicating that they facilitate the quick enhancement of the altered proteins via modifying enzymes. Notably, this procedure saves effort and does not require energy-intensive protein synthesis or breakdown [173]. By controlling the activity, location, and interactions of proteins with nucleic acids and other proteins, PTMs further enhance complexity and variation from the genome to the proteome level. Currently, about 400 different kinds of PTMs are known to exist, regulating various cellular processes [174,175]. For a comprehensive understanding of how protein isoforms and PTMs in plants respond to environmental stresses, we refer to the review by Kosová et al. (2021) [176].

Fast protein function modification in response to changes in the environment and metabolism is made possible by PTMs in proteins. The regulation of light energy distribution between Photosystems I and II (state transitions) and the PSII repair cycle is significantly influenced by phosphorylation. Furthermore, the efficiency of carbon absorption is determined by the thioredoxin-mediated redox control of CBC enzymes. Recent methodological advancements have made it possible to identify many more forms of PTMs in different plant compartments, such as chloroplasts. These modifications affect DNA replication, transcription efficiency, translation machinery regulation, and chloroplast metabolic activities. Furthermore, many PTMs govern the light processes involved in photosynthesis and carbon assimilation at different levels [177]. Phosphorylation, tyrosine nitration, acetylation, lysine methylation, N-terminal processing, and carbamylation are some PTMs that significantly regulate RubisCO, an enzyme essential to carbon fixation [177]. The chaperone RubisCO activase (RCA) is regulated by the ATP/ADP ratio in the chloroplast, as well as the redox state, which is necessary for the reactivation of RubisCO through the carbamylation of lysine in the RubisCO active site [178]. RCA activity increases due to the Fd/Trx system’s reduction of RCA disulfide bridges [179]. To increase photosynthetic activity, activated RCA continually remodels blocked active sites of RubisCO by eliminating inhibitory RuBP [180].

The light-dependent Thr phosphorylation of the PSII core proteins D1 and D2, the inner antenna protein CP43, and a minor PSII subunit, PsbH, is primarily catalyzed by the STN8 kinase. On the other hand, the PSII core phosphatase is responsible for the opposite reaction, dephosphorylation [181]. According to Tikkanen et al. (2008) [182], the phosphorylation of PSII proteins contributes to the folding of the thylakoid membrane, which influences the lateral movement of damaged D1 protein from grana stacks to stroma lamellae for degradation and resynthesis. Additionally, phosphorylation is crucial in balancing electron transport between PSII and PSI in response to external stimuli, such as light quality and quantity [183].

It is already well established that thioredoxin reduces disulfide bonds in specific enzymes of the CBC, which leads to the activation of these enzymes [184]. Apart from redox regulation, several (PTM-dependent) systems meticulously regulate every stage of CO_2_ fixation and starch metabolism. These mechanisms balance the rate of starch synthesis with the availability of energy and carbon in diverse plant tissues and environmental circumstances [177].

RubisCO’s PTMs have been investigated in great detail, and several other enzymes involved in carbon absorption have been demonstrated to contain many PTMs. The stimuli influencing RCA activity are reflected in the yield of the complete carbon-assimilation cycle because RCA is implicated in the onset of carbon fixation by eliminating inhibitors from the RubisCO active center. Stt7 kinase, localized in thylakoids, phosphorylates RCA at Ser53 in green alga *C. reinhardtii*. Although a modest amount of the enzyme has been discovered in connection with the thylakoid membrane, most RCA is located in the stroma [185]. According to certain theories, phosphorylating RCA strengthens its membrane attachment and shields Stt7 from proteolysis [186]. Relocating could also allow RubisCO to operate less in certain environmental circumstances [186]. Several other carbon assimilation-related enzymes undergo PTMs. For example, FBPA is trimethylated at a conserved Lys residue at the protein’s C-terminus. However, this modification does not influence the enzyme’s catalytic activity or oligomeric state [187].

Beyond transcription-translation and allosteric regulation mechanisms, post-translational modifications provide additional control layers that significantly increase the configurations of the plant proteome. Additionally, the PTMs provide dynamic and reversible modifications in subcellular localization and protein function, allowing plants to adjust to environmental changes in a resource-efficient way. PTMs may now be identified with great throughput and accuracy due to new methods and growing computer power. However, obstacles still hinder the discovery of PTMs’ physiological importance. Reverse genetic and in vitro methods must be used to examine the impact of PTMs on protein localization or activity. Furthermore, providing a complete picture of the impacts of PTMs on carbon fluxes is challenging due to the intricacy of carbon metabolism in plants, which involves multiple cellular compartments at the subcellular level as well as long-distance transport and communication at the systemic level. This problem could be resolved in the medium to long future by developing high-throughput gene-editing technologies in conjunction with rapid automated phenotyping devices.

## 4. Improvement in Photosynthesis under Abiotic-Stress Conditions

### 4.1. Under High and Low Light Intensity

In order to survive in nature, plants must be able to react to variations in light intensity that occur over intervals of seconds to minutes. Short-term regulatory systems facilitate a quick transition from a low to a high photosynthetic rate to control these alterations. Furthermore, photosynthesis may be restricted in situations where there is variation in light, particularly when moving from high to low light and vice versa [188]. Regardless of whether there is a rise or decrease in light level or intensity, the amount of time it takes for photosynthesis to return to a steady state after a change in light availability can range from a few minutes to more than thirty minutes, depending on the length and intensity of the shift. Enhancing crop output may, therefore, be possible by adjusting electron transport and the CBC to allow for a rapid response to changes in light availability [132]. For example, excessive light produces toxic oxygen radicals and interferes with the light-harvesting complex (LHC), leading to photoinhibition [189]. Conversely, insufficient light lowers photosynthetic efficiency, increases oxidative stress, and decreases CO_2_ and nitrogen metabolism [190]. Furthermore, low light drastically decreases stomatal conductance efficiency and photosynthesis, which causes the intercellular CO_2_ concentration in leaves to rise quickly [191]. The photosynthetic machinery, stomatal conductance, maximum quantum efficiency of PSII, transpiration rate, and average photosynthesis rate are thereby inhibited by low-light stress [192].

The photosynthetic machinery is also harmed by extreme light stress. For instance, plants in tropical climates experience high temperatures and high light intensities, which reduces photochemical efficiency and excites photons in the chloroplast [193]. While PSI’s performance is not greatly impacted in these circumstances, PSII’s efficiency declines [194]. When the intrinsic mechanism responsible for D1 protein repair is overwhelmed by oxidative damage under high light intensity, the degradation in the PSII reaction center leads to photoinhibition [57]. Furthermore, electron transport, photochemical efficiency, and photo-oxidation under high light stress and high temperature further impede PSII’s quantum efficiency. Strong light intensity causes chlorophyll b (Chl b) breakdown via the Chl b reductase enzyme [195]. In another study, high light intensity was found to damage *Dunaliella salina* cells, as shown by research by Xu et al. (2016), where free hydroxyl radicals oxidize PSI, leading to photoinhibition [196].

Pospisil et al. (2016) found that O_2_’s interaction with the photosynthetic electron transport chain (ETC) controls the formation of ROS during photosynthesis. ROS generation rises in abundant light, and plants use antioxidant enzymes, detoxification processes, and repair mechanisms to prevent ROS from building up to dangerous levels [197,198]. Furthermore, strong light-intensity-induced ROS generation causes plant cell death [199]. By regulating PSII’s photoinhibition in extreme light stress, the prenyl lipid plastoquinone-9, which acts as an electron transporter between PSII and PSI, safeguards the photosynthetic machinery [200].

Previous research has examined the role of several transcription factors in the direct or indirect control of genes associated with photosynthesis through hormone pathways. The effectiveness of the PSII system has been linked to the regulation of GhDREB and CRF6, as well as the amount of chlorophyll. Plant transcription factors, including BZR1 and WRKY, have been observed to affect genes linked to the chloroplast and cell wall [189]. Stress tolerance critically depends on controlling CAB gene expression via the light-responsive LONG HYPOCOTYL 5 (HY5) and bZIP-type transcription factors [201]. According to Agarwal et al. (2017) [202], the C-repeat binding factor/dehydration-responsive element (CRT/DREB)-binding transcription factor family actively organizes signal transduction networks, senses environmental stimuli, and regulates the expression of vital genes. The most significant function of CBF/DREB transcription factors is the regulation of all co-regulated genes, which enhances plant growth and development by preserving normal photosynthesis, stomatal conductance, chlorophyll content, and ETC efficiency under stressful circumstances.

In addition to the well-established function of transcription factors, several candidate genes have also been identified as quick and dependable means of directly connecting leaf photosynthesis in agricultural plants to their tolerance of various abiotic stressors. For instance, in rice, the overexpression of the Arabidopsis HARDY (HRD) gene lowered the transpiration rate and increased photosynthetic absorption [203]. ABR17, a protein belonging to the pathogenesis-related protein (PR10) family, enhanced Arabidopsis seed germination rates and showed enhanced tolerance against various stress stimuli [204]. Mitogen-activated protein kinases (MAPKs) are protein kinases that play a variety of roles in eukaryotic cells, including metabolic and cellular processes, response to external stimuli, and chloroplast-redox-regulated gene expression under stress conditions. These roles collectively enhance the photosynthetic rate to optimal levels [205].

### 4.2. Under High Temperatures (Heat Stress)

Increasing global temperatures is one of the main issues currently influencing plant viability. A temperature increase over a particular threshold reduces the net yield and impairs cellular homeostasis and plant metabolism [206,207]. Among the two photosystems, PSII is the more sensitive part of the photosynthetic apparatus to high-temperature stress and is the main site where photoinhibition occurs [208,209]. Under high-temperature stress, due to the enhanced fluidity of the thylakoid membrane, the PSII light-harvesting complex easily detaches from the thylakoid membrane, destroying the integrity of PSII and affecting photosynthetic electron transfer [210]. High-temperature stress has an inhibitory effect on the oxygen-evolving complex (OEC) [211], the reaction center, and the electron acceptor side of PSII [212]. If the damage rate of PSII exceeds the repair rate, it leads to a reduction in light energy-utilization efficiency, resulting in photoinhibition [129]. During high-temperature stress, the inability to promptly dissipate excess excitation energy in the photosynthetic mechanism leads to ROS accumulation. This accumulation directly damages the PSII reaction center protein and impedes the PSII repair process by de novo synthesizing the D1 protein, intensifying PSII photoinhibition [129]. Compared with PSII, PSI is less sensitive to moderate high-temperature stress. Studies have found that high-temperature stress increases the reduction rate of P700+ in the PSI reaction center [208,213], indicating that high temperatures promote the electron transfer rate of PSI. Under natural conditions, plants are often in an environment with fluctuating light intensity. The rapid increase in light intensity on the leaf surface causes the electrons transferred from PSII to PSI to not be consumed immediately, resulting in an excessive reduction of PSI [214]. During this time, high-temperature stress can cause PSI photoinhibition [215]. Once photoinhibition occurs in PSI, it often has a greater impact on photosynthesis due to the longer recovery time needed for PSI compared to PSII [216].

In addition, high temperature can affect photosynthetic electron transfer by impeding the efficient flow of electrons within the photosynthetic process, further contributing to the overall disruption of the photosynthetic apparatus. Under normal conditions, there are at least two electron-transfer pathways in plant photosynthesis: linear electron flow (LEF) driven by PSII, the PQ pool, the Cyt b6/f complex, and PSI [217]; and cyclic electron flow (CEF) driven only by PSI [218,219]. Reducing LEF under adversity stress can prevent excess electrons from being transferred from PSII to PSI, thereby alleviating the accumulation of ROS in PSI and protecting PSI from high-temperature damage [220,221]. In photosynthetic electron transport, the PQ pool plays a functional connection role between PSII and the Cyt b6/f complex, and its redox state can effectively regulate photosynthetic reactions, including regulating state transitions, chlorophyll biosynthesis, and photosystem protein synthesis rates [222,223]. Due to high-temperature stress causing damage to the OEC, the proportion of the oxidized PQ pool in leaves increases [224]. The oxidized PQ pool can effectively quench the excited chlorophyll molecules in the PSI antenna [225], thus protecting PSI.

Studies have found that the important physiological functions of CEF include participation in the induction of NPQ, the redox regulation of the PSI complex, and the regulation of the ATP/NADPH ratio [226,227]. Under stress, CEF is the main source of ΔpH formation in chloroplasts [228]. Under high-temperature stress, plants activate CEF and dissipate heat with higher qE by promoting ΔpH [229]. Additionally, there is evidence that H_2_O_2_ may serve as a connecting factor between environmental stress and CEF induction, playing a role in activating CEF [230]. Ca^2+^ also plays an important role in this process. Based on previous research results, it is speculated that high-temperature stress mainly leads to the production of ROS by causing the accumulation of excess light energy. ROS then serve as a signaling molecule to induce CEF activity through the Ca^2+^ signaling cascade reaction [231].

In addition, the thermal instability of the enzymes directly involved in photosynthesis is a key reason for the decrease in photosynthetic rate under temperature stress [232]. At the biochemical level, the net photosynthetic rate of plants largely depends on the activation and activity of RubisCO and RuBP. RubisCO is a bifunctional enzyme, and high-temperature stress exceeding 35 °C can inhibit its initial carboxylation activity in rice leaves. SBPase is a key enzyme in the Calvin cycle. The overexpression of SBPase can improve the ability of rice to withstand high-temperature stress, indicating that increasing SBPase to provide RuBP to RubisCO can enhance the plant’s stress resistance [233]. Transketolase, a thiamine pyrophosphate-dependent enzyme, not only regulates carbon fixation in higher plants but also responds to abiotic stress. As RubisCO’s specificity for CO_2_ and O_2_ decreases with increasing temperature, the ratio of carboxylation to oxidation reactions diminishes. Consequently, RubisCO tends to oxidize under high-temperature stress, producing more 2-phosphoglycolic acid and entering the photorespiration pathway, which consumes energy and leads to a loss of photosynthetic carbon fixation [234]. Furthermore, RubisCO activity is regulated by RCA [235]. Studies have shown that RCA possesses ATPase activity and can release sugar phosphate inhibitors from RubisCO’s active site, thereby activating the enzyme [236]. The RCA-activation process depends on ATP and is inhibited by ADP [237]. RCA activity is easily inhibited by high-temperature stress, and its gene expression is sensitive to short-term heat stress [238]. Wang et al. (2020) [239] reported an isoform-specific difference in the RCA forms in Rhododendron hainanense, with the novel heat-induced RCA1 showing higher expression and greater thermostability under heat stress compared to the constitutively expressed RCA2 and RCA3. Therefore, improving the thermostability of RCA has become an important strategy to alleviate the impact of high-temperature stress on photosynthetic capacity and crop yield.

In addition, high temperature stress can easily cause the total chlorophyll content of leaves to decrease [240]. As the main photosynthetic pigment on the thylakoid membrane of chloroplasts, chlorophyll plays a critical role in capturing and driving photosynthetic electron transfer. Under normal conditions, the synthesis and degradation of chlorophyll are balanced. However, in a high-temperature environment, the activity of 5-aminovaleronate dehydratase, the first enzyme involved in the pyrrole synthesis pathway, is inhibited [241]. Concurrently, the activity of chlorophyllase and peroxidase, which are involved in chlorophyll degradation, is increased [242]. This imbalance results in reduced chlorophyll content and accelerated leaf senescence. Since chlorophyll captures a significant amount of light energy, its degradation under high temperatures can be seen as a protective response to prevent excess light energy from damaging the photosynthetic machinery.

### 4.3. Under Low Temperature (Cold Stress)

Plants under cold stress have a reduced absorption of nutrients and water, resulting in cell starvation. To withstand the cold, plants generate a variety of proteins. Low temperatures also impact the photosynthetic rate (Pn) of plants, leading to a decrease in biomass. This reduction in photosynthetic rate is attributed to two mechanisms: stomatal limitation and non-stomatal limitation. If both stomatal conductance (Gs) and the intercellular CO_2_ concentration (Ci) decrease simultaneously over time, the decline in Pn is due to stomatal-limiting factors. Conversely, if Gs decreases over time while Ci increases, non-stomatal factors are responsible for the decrease in Pn [243].

Conditions leading to low-temperature photoinhibition in cold-sensitive plants encompass a range of factors, including low temperatures (0~10 °C), reduced oxygen levels, weak light, prolonged exposure to low temperatures, and the typical electron flow from PSII. Under normal conditions, PSII exhibits a standard current effect, but at low temperatures, it inhibits the current carrier, diminishing transfer carrier efficiency and reducing the proton gradient. Previous research predominantly focused on PSII, pinpointing it as the site of photoinhibition. However, recent studies propose that PSI is more susceptible to low temperatures, displaying a greater likelihood of photoinhibition effects [244]. PSI photoinhibition primarily results from the detrimental effects of ROS. Cold-sensitive plants, like cucumbers, generate peroxide or singlet oxygen under low-temperature and low-light conditions, causing damage to the PSI reaction center. The deactivation of the reactive oxygen defense system exacerbates photodamage [245]. The reducing side of PSI produces peroxide, and enzymes from the reaction center scavenge ROS. As temperatures drop, the CO_2_ assimilation rate decreases, causing a continuous increase in the reducing power of the PSI receptor side. Upon recovery, the PSI receptor side generates triplet P700, which reacts with oxygen to produce singlet oxygen, leading to photoinhibition [246].

Thylakoid electron transfer (TET), the transpiration rate, stomatal conductance, and the carbon-reduction cycle are among the photosynthetic characteristics impacted by low or freezing temperatures [247]. To withstand cold conditions, plants have developed novel defense mechanisms. During this process, plants produce protective chemicals, such as proline and soluble sugars, and proteins like late embryogenesis abundant (LEA), which have higher freezing tolerance and resilience to cold stress [248]. Similar biochemical reactions occur in response to cold and freezing stress as in the response to drought and salt stress. These reactions include disrupting redox homeostasis, leading to the increased production of ROS and the increased synthesis of enzymatic and non-enzymatic antioxidants. Additionally, plants produce pathogen-related (PR) proteins, heat-shock proteins (HSPs), and sugar alcohols to combat stress conditions [249].

In addition to the buildup of soluble sugars in cells, the concentration of polyunsaturated fatty acids in membranes rises during low-temperature adaptation to preserve appropriate membrane fluidity [250]. Crops with increased cold tolerance have been developed through metabolic engineering utilizing these physiological processes. For instance, increased freezing tolerance was obtained in transgenic tobacco by overexpressing the plastidial α-3 fatty acid desaturase gene, which is involved in manufacturing linolenic acid [251]. Similarly, in transgenic plants, the overexpression of the glycerol-3-phosphate acyl transferase gene altered the unsaturation of fatty acids, conferring added cold tolerance [252]. Furthermore, improved stress tolerance was observed in transgenic rice that expressed cold shock protein (CSP) genes from bacteria under various abiotic stress conditions, such as cold, heat, and water deficiencies [253,254]. Additionally, it has been demonstrated that rice’s ability to withstand cold is enhanced by RAN1, a member of the G-protein family [255]. 

According to Sanghera et al. (2011), transcription factors are a promising target for improving crop plants’ resistance to freezing and cold [254]. When overexpressed under appropriate promoters, genes encoding DREB1/CBF, which are cold-inducible transcription factors, also confer resistance to salt and drought [256]. This has been demonstrated in rice and Arabidopsis. Plants overexpressing CBF3 exhibited a higher freezing tolerance, as evidenced by the constitutive accumulation of cold acclimation response (COR) proteins and the increased accumulation of proline and sucrose [257,258]. In transgenic tobacco and Arabidopsis, the overexpression of the zinc finger protein SCOF-1 gene led to increased resistance to low temperatures [259]. 

### 4.4. Under Drought Stress

Droughts are becoming more prevalent globally due to global warming and decreased subsurface water supply, significantly impacting photosynthesis and crop production [260,261]. A shortage of water reduces chlorophyll levels, severely damages the thylakoid membrane, and restricts the effectiveness of the photosynthetic machinery [62]. Droughts also lower the quantum yield of PSII, which has detrimental effects [262]. Studies indicate that drought stress reduces turgor pressure in plants, inhibiting growth, biomass, and shoot length [263]. Water deprivation substantially impacts the photosynthetic machinery; for instance, reduced water content causes the degradation of the thylakoid membrane and chlorophyll pigments [264]. To survive such hostile conditions, plants activate their defense mechanisms and eventually self-adjust through various strategies, including stomata closure, osmotic adjustment, and elevated tolerance levels [265].

To develop drought-tolerant crops, dehydration-responsive element-binding (DREB) transcription factors from ABA-independent pathways are attractive targets, provided that suitable tissue-specific, drought-inducible promoters are utilized to prevent undesirable side effects. Transgenic crops such as wheat, soybeans, tobacco, and tomatoes have shown improved drought tolerance when genes encoding DREB/CBF are overexpressed [266]. However, Nakashima et al. (2014) noted that DREB2s are involved in both heat-shock and dehydration responses [256]. Under field conditions, the overexpression of the genes encoding NAM, ATAF, and CUC transcription factors improved rice’s resilience to drought and its ability to withstand salinity [267]. Additionally, transgenic lines with enhanced photosynthetic rates showed improved drought tolerance when the genes encoding the kinase domain of NPK1, a tobacco MAPK kinase, were constitutively overexpressed in maize [268]. Xiao et al. (2009) reported that transgenic rice plants exhibited increased grain production under drought-stress conditions when the LOS5/ABA3 gene, coding for the main enzyme of ABA biosynthesis, was overexpressed [269]. Furthermore, improved drought tolerance was seen in transgenic rice by overexpressing a gene producing P5CS, the rate-limiting enzyme in proline biosynthesis [270]. 

A MnSOD gene was cloned from the drought-tolerant Chinese shrub *Tamarix androssowii* and inserted into cotton. The resulting transgenic lines displayed improved physiological performance and increased total biomass under drought stress [271]. According to Liu et al. (2021), recent developments in nano-biotechnology have the potential to significantly increase agricultural production by strengthening plant tolerance processes [272]. Mahmoud et al. (2020) reported that SiO_2_ nanoparticles (SiO_2_-NPs) can mitigate drought stress in banana plants [273]. SiO_2_-NPs facilitate photosynthesis and reduce cellular damage in stressed plants by regulating ion balance and ROS metabolism [274]. Enhancing drought-induced photosynthetic inhibition can be achieved by sequestering nanoceria, a negatively charged polyacrylic acid nanomaterial, inside chloroplasts [275]. Research using the magneto-priming method to quickly incorporate SiO_2_-NPs into seeds has shown improvements in crop production, crop establishment, and seedling establishment in water-stressed sesame [276]. Additionally, SiO_2_-NPs have been demonstrated to improve cucumber growth and yield under saline and water-deficit conditions by increasing mineral translocation, controlling the Na/K ratio, and increasing silicon translocation to the leaves. These actions help to regulate osmotic balance by maintaining ion homeostasis, managing stomatal gas exchange and water flow in the soil-plant-atmosphere system, and enhancing photosynthesis in stressed plants, ultimately leading to elevated yield [277]. 

### 4.5. Under Salt Stress (Salinity)

Hossain and Dietz (2016) [278] reported that transgenic plants overexpressing genes encoding antioxidant enzymes such as GST/GPX, APX, SOD, and CAT or having an increased concentration of non-enzymatic antioxidants demonstrated higher salinity tolerance. Guo et al. (1997) found that, in rice, glycine betaine levels and salt tolerance were both enhanced by the expression of the *Atriplex hortensis* BALDH gene (betaine aldehyde dehydrogenase) [279]. Recently, it has been demonstrated that citrus plants stressed by salt and drought significantly express genes of the tonoplast intrinsic protein (TIP) isoform (CsTIP2;1) aquaporin [280]. In transgenic tobacco, the overexpression of this gene promoted plant growth in normal and drought- and salt-stressed environments. Furthermore, plants exposed to soil dryness showed considerable improvements in photosynthetic capability, transpiration rate, water usage efficiency, and leaf water and oxidant status when treated with CsTIP2;1 [280]. Transgenic plants exhibiting tolerance to salinity stress often also show resistance to other stressors, including drought [281] and cold stress [254]. Broad abiotic stress resistance is specifically conferred by the enhanced synthesis of trehalose, CIPK, or DREB transcription factors [282].

Plant viability is reduced when roots receive less water and have decreased activity in their water channels due to salinity stress [283]. To mitigate oxidative stress, plants employ various strategies, including enhancing and sustaining antioxidant activity and upregulating specific gene expression in response to salinity. The identification of many plant aquaporins (AQPs) in roots suggests that these molecules may be activated in response to various environmental stimuli and may have specialized roles in various cell types [284]. Kumawat et al. (2021) describe the AQP gene family as multifunctional channels that transport several substances, including water, metalloids, CO_2_, and hydrogen peroxide [285]. 

Nanonutrients are nutrients engineered at the nanoscale, typically less than 100 nanometers, which enhance nutrient delivery and effectiveness in biological systems. These nanoscale nutrients offer improved absorption and bioavailability, controlled release, and targeted delivery, leading to more efficient nutrient use and reduced environmental impact. This innovative approach is particularly beneficial in agriculture. Nanonutrients for agriculture are designed to be released slowly and steadily, matching the nutrient demands of crops over an extended period, usually more than 30 days. This controlled release can enhance the efficiency of nutrient use and potentially reduce environmental pollution compared to conventional fertilizers [286,287]. According to Tripathi et al. (2015), SiO_2_-NPs can boost the photosynthetic system and increase chlorophyll synthesis [288]. TiO_2_-NPs have been shown to boost photosynthetic pigment content and activity in salt-stressed garlic (*Allium sativum*) plants compared to control plants [289]. TiO_2_ efficiently absorbs light, providing energy to electrons, and increases the activity of the RubisCO enzyme, which is central to the CBC, thereby increasing carbon absorption [290]. Almodares et al. (2007) noted that salt stress inhibits growth by creating osmotic barriers that reduce seeds’ ability to absorb water and cause toxicity in seed embryos [291]. Under NaCl stress, SiO_2_-NPs significantly increase chlorophyll content and promote seed germination [292]. Soil salinity and dryness are major stressors affecting agricultural productivity in semi-arid and arid environments. However, NPs have demonstrated the ability to support irrigated crops like rice in these places, increasing their growth and yield [293,294]. 

Badawy et al. (2021) investigated the impact of nanonutrient-loaded nanoparticles on shunted growth patterns in Oryza sativa L. under saline stress. The highest relative water content and dry matter were obtained from soaking rice grains in silicon (Si)- and selenium (Se)-NPs. In contrast, the maximum leaf area index was obtained from foliar-spraying Se-NPs during the booting stage, compared to other treatments. By improving ion homeostasis in plant cells, Si-NPs and Se-NPs as nanonutrients helped to reduce the harmful effects of salinity and increase grain output in rice crops [295]. The selected genes summarized in this review, categorized by their respective roles in responding to abiotic stress, are listed in Table 2.

## 5. Future Perspectives

Enhancing photosynthetic efficiency through genetic engineering presents several major challenges. Photosynthesis is a crucial metabolic process that has evolved over millions of years and is deeply integrated into the overall metabolism of photosynthetic organisms. For instance, the metabolites produced during photorespiration influence other processes, such as nitrate assimilation, which may not seem directly related. Thus, altering one part of the photosynthetic machinery invariably impacts other metabolic reactions, and our understanding of the integration between metabolic and regulatory pathways remains limited. 

To address this complexity, multi-target genetic modifications are needed, involving the simultaneous manipulation of multiple enzymes and regulatory proteins within the CBC and other pathways. System biology can help develop more complex models that predict target genes. This integrated approach can prevent metabolic imbalances and maximize photosynthetic efficiency. Another significant limitation arises from the methodologies currently available. Photosynthetic traits are governed by genes expressed in both nuclear and plastid genomes, necessitating highly effective transformation methods for both genomes to achieve successful genetic modifications. Advanced synthetic biology techniques should be leveraged to design and construct novel metabolic pathways to enhance carbon fixation and reduce energy losses. Additionally, optimizing components of the ETC by reintroducing lost evolutionary elements could significantly boost photosynthetic rates. Focusing on stress-resilient photosynthesis by investigating stress-responsive genes and regulatory mechanisms can provide valuable insights. These genetic modifications can improve the plants’ antioxidant capacity and protect photosynthesis under adverse conditions.

Another promising area is exploring the genetic variation in wild species to uncover novel genes and alleles associated with improved photosynthesis. High-throughput phenotyping and genome-wide association studies can facilitate the identification and introgression of these beneficial traits into commercial cultivars. Several efforts have attempted to gain insight into natural variation; however, photosynthesis traits are highly sensitive to the environment. Therefore, more robust phenotyping are required [296,297,298,299]. 

The emerging field of nanotechnology presents a promising avenue for enhancing photosynthesis by facilitating more efficient nutrient delivery, optimizing light absorption through engineered nanomaterials, and protecting photosynthetic machinery from environmental stress. Additionally, integrating nanotechnology into gene-modification strategies allows for highly precise and controlled gene manipulation. By enabling the targeted delivery of genetic materials or regulatory molecules directly to specific cells or organelles, it also reduces off-target effects, ensuring more efficient gene expression and creating new possibilities for enhancing photosynthesis. By pursuing these research directions, the scientific community can develop crops with superior photosynthetic efficiency and resilience, contributing to sustainable agriculture and global food security.

## 6. Conclusions

The ongoing quest to enhance photosynthesis through genetic engineering provides exciting opportunities to increase crop yields and address global food security challenges. This review underscores the complexity of the genetic basis of photosynthesis and highlights various successful interventions aimed at improving it. Major achievements include the engineering of photosynthetic pathways—such as the CBC, electron transport chain, and photorespiration using gene-editing tools like CRISPR-Cas9 and synthetic biology. Additionally, genetic engineering to increase plants’ resilience to environmental stresses such as temperature and drought has shown significant potential. These strategies not only boost photosynthetic efficiency but also enhance plant growth and biomass production under fluctuating environmental conditions. Further research should aim to integrate these genetic enhancements to improve photosynthetic efficiency and stability, ultimately fostering a sustainable and food-secure future.

## Figures and Tables

**Table 1:** List of key genetically engineered genes for enhancing photosynthetic pathway efficiency.

Gene	Function and Impact	Pathway
**Target: Thylakoid membrane**		
PsbS	Regulates non-photochemical quenching (NPQ) to enhance photoprotection, preventing damage from excess light during photosynthesis.	Non-photochemical quenching (NPQ)
PGR5/PGRL1	Regulates cyclic electron flow (CEF) to protect photosystem I (PSI), enhancing the plant’s resilience to fluctuating light conditions.	Cyclic electron flow
KEA3	Regulates the proton gradient across the thylakoid membrane, optimizing ATP synthesis and overall photosynthetic efficiency.	Proton gradient regulation
VDE (violaxanthin de-epoxidase)	Converts violaxanthin to zeaxanthin in NPQ, playing a critical role in dissipating excess light energy as heat.	Non-photochemical quenching (NPQ)
ZEP (zeaxanthin epoxidase)	Converts zeaxanthin back to violaxanthin, regulating NPQ and maintaining balance in the light-harvesting process.	Non-photochemical quenching
Reisek FeS protein	Mediates electron flow between PSII and PSI, playing a pivotal role in the overall efficiency of the electron transport chain.	Electron transport chain
NADH dehydrogenase (NDH)	Involved in cyclic electron flow around PSI, contributing to ATP production and enhancing the efficiency of photosynthesis.	Cyclic electron flow
**Target: Chloroplast**		
SBPase (sedoheptulose-1,7-bisphosphatase)	Enhances the regeneration of ribulose bisphosphate (RuBP) in the Calvin-Benson cycle, facilitating increased carbon fixation.	Calvin-Benson cycle
CAO (chlorophyllide a oxygenase)	Modulates chlorophyll b levels to optimize light capture, improving overall photosynthetic efficiency by enhancing light harvesting.	Light-harvesting complex
FBPA (fructose-1,6-bisphosphate aldolase)	Improves photosynthesis and growth under elevated CO_2_ conditions by facilitating the conversion of sugars in the Calvin cycle.	Calvin-Benson cycle
GAPDH (glyceraldehyde-3-phosphate dehydrogenase)	Increases the production of glyceraldehyde-3-phosphate (G3P) in the Calvin-Benson cycle, enhancing carbohydrate synthesis and energy production.	Calvin-Benson cycle
PRK (phosphoribulokinase)	Crucial for the regeneration of RuBP; overexpression can significantly improve photosynthetic rates and biomass accumulation.	Calvin-Benson cycle
RCA (RubisCO activase)	Reactivates RubisCO, ensuring efficient carbon fixation by maintaining the enzyme’s activity under varying conditions.	Calvin-Benson cycle
Ferredoxin-NADP+ reductase (FNR)	Reduces NADP+ to NADPH, a critical step for providing the reducing power necessary for the Calvin cycle.	Electron transport chain
**Target: Mitochondria**		
Glycine decarboxylase (GDC)	Involved in the photorespiratory pathway, mitigating the effects of photorespiration and enhancing overall carbon efficiency.	Photorespiration
H-protein	Enhances photorespiration by improving glycine cleavage system (GCS) efficiency, facilitating better nitrogen assimilation.	Photorespiration
T-protein	Part of the GCS, crucial for facilitating the conversion of glycine during photorespiration, thereby affecting overall carbon balance.	Photorespiration
**Target: Nucleus**		
GLK transcription factors	Enhances photosynthesis by regulating chloroplast development, promoting the formation of functional chloroplasts necessary for light capture.	Chloroplast development
psbA	Involved in the de novo synthesis of the D1 protein, essential for the repair of photosystem II (PSII) under photoinhibition conditions.	Photoinhibition
mEmBP1	Enhances photosynthesis by regulating chloroplast development, similar to GLK, ensuring efficient light utilization.	Gene regulation
HYR (higher yield regulation)	Acts as a master regulator for photosynthesis genes, coordinating responses to environmental signals and optimizing growth.	Gene regulation
NRP1 (Negative Regulator of Photosynthesis 1)	Negatively regulates photosynthesis-related genes, providing a balance in the expression of photosynthesis-related pathways under stress.	Gene regulation

**Table 2:** A summary of key genes and proteins involved in enhancing plant resistance to various abiotic stresses, their roles, and the organisms in which they have been studied.

Gene	Organism	Role/Function	References
**Abiotic Stress: High Light Intensity**			
**GhDREB, CRF6**	Plants	Regulates the PSII system, chlorophyll content, and stress tolerance	[189]
**BZR1, WRKY**	Plants	Affects genes linked to the chloroplast and cell wall	[189]
**HY5, bZIP-type TFs**	Plants	Regulates CAB gene expression, enhancing stress tolerance	[201]
**HARDY (HRD)**	Arabidopsis/rice	Lowers the transpiration rate and increases photosynthetic absorption	[203]
**ABR17**	Arabidopsis	Enhances seed germination rates and stress tolerance	[204]
**MAPKs**	Eukaryotic cells	Enhances the photosynthetic rate by regulating chloroplast redox and gene expression	[208,209]
**Abiotic Stress: High Temperature**			
**RubisCO activase (RCA)**	Rice	Activates RubisCO, improving thermal stability and photosynthetic capacity under heat stress	[238]
**Sedoheptulose 1,6-bisphosphatase (SBPase)**	Rice	A key enzyme in the Calvin cycle; it enhances high-temperature stress resistance	[233]
**RAN1**	Rice	A member of the G-protein family; it enhances cold stress tolerance	[255]
**Abiotic Stress: Low Temperature**			
**CBF/DREB1**	Rice, Arabidopsis	Confers resistance to cold, drought, and salt stress by regulating the cold acclimation response	[256,257]
**SCOF-1**	Tobacco, Arabidopsis	A zinc finger protein; it increases resistance to low temperatures	[259]
**CSPs**	Bacteria/Plants	Enhances cold, heat, and water-deficiency tolerance	[253,254]
**Plastidial α-3 fatty acid desaturase**	Tobacco	Is involved in linolenic acid synthesis; it improves freezing tolerance	[251]
**Glycerol-3-phosphate acyl transferase**	Plants	Changes fatty acid unsaturation; it enhances cold tolerance	[252]
**Abiotic Stress: Drought Stress**			
**DREB/CBF**	Various species	Improves drought tolerance by regulating stress-responsive genes	[266]
**NAM, ATAF, CUC**	Rice	Enhances drought and salinity resistance	[267]
**NPK1**	Maize	Kinase domain; it improves drought tolerance	[268]
**LOS5/ABA3**	Rice	A key enzyme in ABA biosynthesis; it enhances drought tolerance	[269]
**P5CS**	Rice	A rate-limiting enzyme in proline biosynthesis; it improves drought tolerance	[270]
**MnSOD**	Cotton	An antioxidant enzyme; it enhances drought tolerance	[271]
**Abiotic Stress: Salt Stress**			
**GST/GPX, APX, SOD, CAT**	Various species	Antioxidant enzymes; they enhance salinity tolerance	[278]
**BALDH**	Rice	Enhances the glycine betaine level and salt tolerance	[279]
**TIP (CsTIP2;1)**	Citrus, Tobacco	Aquaporin; it enhances growth and photosynthetic capabilities under salt and drought stress	[280]
**Trehalose, CIPK, DREB TFs**	Various species	Broad abiotic stress resistance	[282]
**AQP gene family**	Plants	Supports water and solute transport and enhances salinity tolerance	[284]

## Supplementary Materials

The following supporting information can be downloaded at: https://www.mdpi.com/article/10.3390/ijms25168933/s1.
