# Supplementary material for: Photosynthesis: Genetic Strategies Adopted to Gain Higher Efficiency
Source: Int J Mol Sci. 2024 Aug 16;25(16):8933. doi: 10.3390/ijms25168933 (PMC11355022; doi:10.3390/ijms25168933)
Supplement: Supplementary file 1 [file ijms-25-08933-s001.zip › ijms-3124217-supplementary.pdf]

Photosynthesis: Genetic Strategies Adopted to Gain Higher Efficiency

Naveed Khan [1,2,†], Seok-Hyun Choi [1,†], Choon-Hwan Lee [2,3], Mingnan Qu [4,*] and Jong-Seong Jeon [1,*]

1 Graduate School of Green-Bio Science, Kyung Hee University, Yongin 17104, Republic of Korea
2 Life and Industry Convergence Research Institute, Pusan National University, Miryang 50463, Republic of Korea
3 Department of Molecular Biology, Pusan National University, Busan 46241, Republic of Korea
4 Jiangsu Key Laboratory of Crop Genomics and Molecular Breeding, College of Agriculture, Yangzhou University, Yangzhou 225009, China
* Correspondence: qmn@yzu.edu.cn (M.Q.); jjeon@khu.ac.kr (J.-S.J.)
† These authors contributed equally to this work.

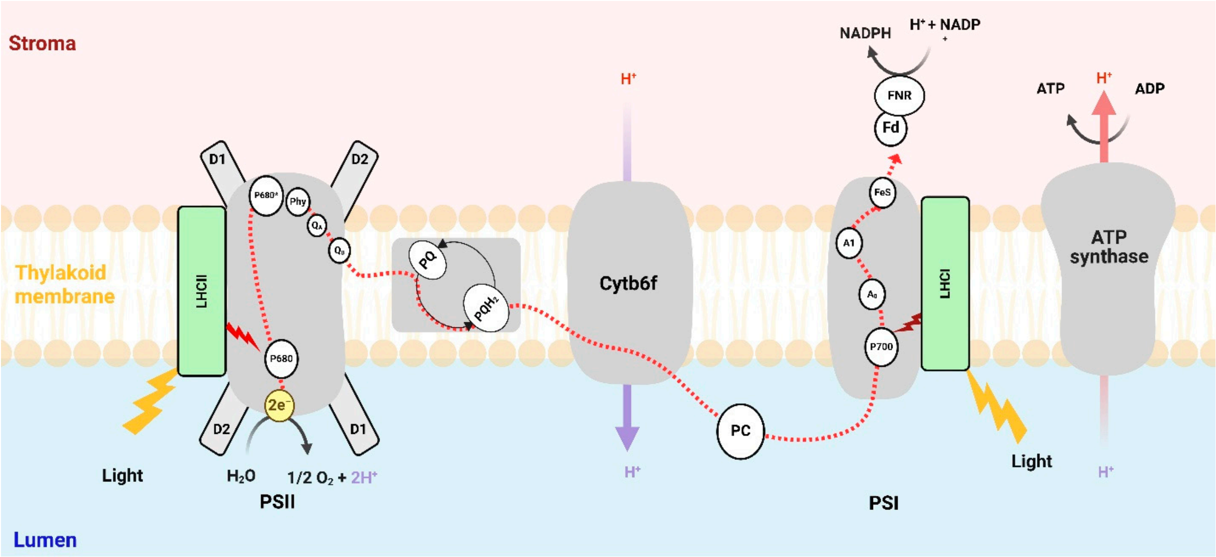

Supplementary Figure S1. A classical schematic diagram of the linear electron transfer LET.

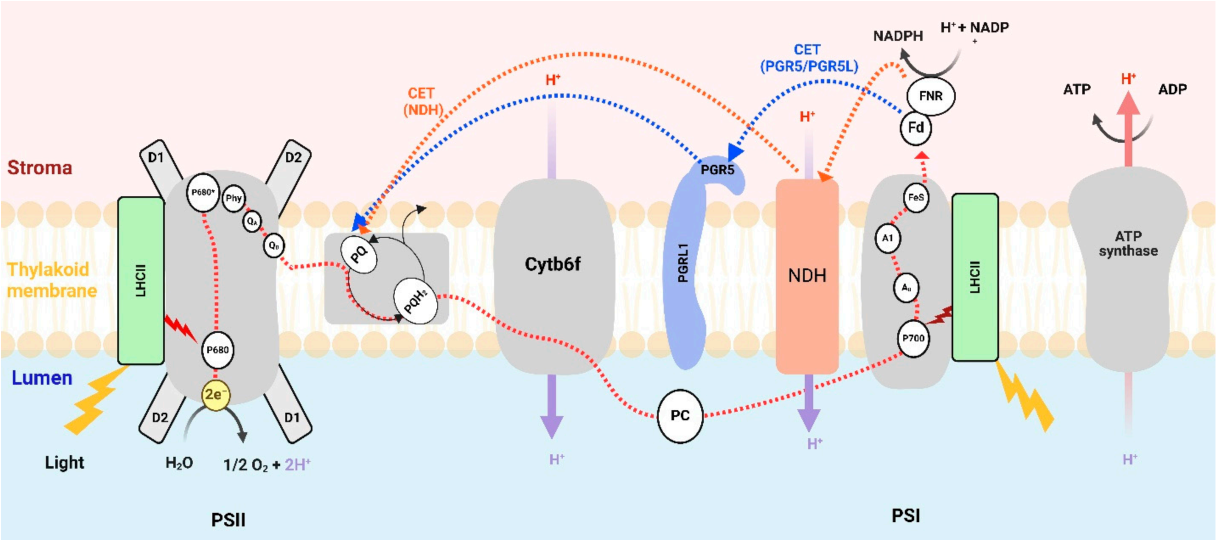

Supplementary Figure S2. This figure shows typical linear electron transfer (LET) and cyclic electron transfer CET.
